# Supplementary material for: Stable Isotopomers of myo-Inositol Uncover a Complex MINPP1-Dependent Inositol Phosphate Network
Source: ACS Cent Sci. 2022 Dec 5;8(12):1683–94. doi: 10.1021/acscentsci.2c01032 (PMC9801504; doi:10.1021/acscentsci.2c01032)
Supplement: Supplementary file 3 — oc2c01032_si_003.pdf [file oc2c01032_si_003.pdf]

Supporting Information

Stable isotopomers of *myo*-inositol uncover a complex MINPP1-dependent inositol phosphate network

Minh Nguyen Trung[1,2], Stefanie Kieninger[3], Zeinab Fandi[1], Danye Qiu[4], Guizhen Liu[4], Neelay K. Mehendale[1], Adolfo Saiardi[5], Henning Jessen[4], Bettina Keller[3], Dorothea Fiedler[1,2*]

Affiliations

[1] Leibniz-Forschungsinstitut für Molekulare Pharmakologie, Robert-Rössle-Straße 10, 13125 Berlin, Germany

[2] Institut für Chemie, Humboldt-Universität zu Berlin, Brook-Taylor-Straße 2, 12489 Berlin, Germany

[3] Institut für Chemie und Biochemie, Freie Universität Berlin, Arnimallee 22, 14195 Berlin, Germany

[4] Institut für Organische Chemie, Albert-Ludwigs-Universität Freiburg, Albertstraße 21, 79104 Freiburg, Germany

[5] MRC Laboratory for Molecular Cell Biology, University College London, WC1E 6BT London, UK

* corresponding author: fiedler@fmp-berlin.de

Table of Contents

Abbreviations 3

Supporting Figures and Tables 4

Experimental section 18

- Safety statement 18
- General Information 18
- NMR data acquisition and processing 18
- CE-MS measurement 18
- Data handling 19
- Synthesis of ^{13}C-labeled Ins and InsPs 19
  - Chemoenzymatic synthesis of *myo*-inositol isotopomers 20
  - Synthesis of 1[$^{13}C_1$]InsP$_6$ 21
- Cloning, expression and purification of recombinant human MINPP1 23
- NMR-based enzymatic assays 25
- Malachite green-based enzymatic assays 25
- Mammalian cell culture and metabolic labeling 25
- Subcellular organelle isolation 26
- Western blots 27
- References 28
- NMR spectra 30

Abbreviations

ACN	acetonitrile
BIRD	bilinear rotation decoupling
BIRD-HMQC	HMQC with BIRD pulse
BPG	2,3-bisphoshpoglycerate
CD	circular dichroism spectroscopy
CE-MS	capillary electrophoresis electrospray mass spectrometry
4,5-DCI	4,5-dicyanoimidazole
DCl	deuterium chloride
DCM	dichloromethane
DMEM	Dulbecco’s Modified Eagle Medium
DMSO	dimethylsulfoxide
DTT	dithiothreitol
EDTA	ethylenediaminetetraacetic acid
FBS	fetal bovine serum
GndHCl	guanidium hydrochloride
GroPI	glycerophosphoinositol
HMQC	heteronuclear multiple-quantum correlation
Ins	*myo*-inositol
InsPx	inositol phosphate
IPS	inositol phosphate synthase
IPTG	isopropyl *β*-D-1-thiogalactopyranoside
MINPP1	multiple inositol polyphosphate phosphatase 1
MWCO	molecular weight cut-off
NAD^+	nicotinamide adenine dinucleotide (oxidized form)
NaOD	sodium deuteroxide
NMR	nuclear magnetic resonance (spectroscopy)
OD_{600}	optical density (at 600 nm)
ORF	open reading frame
PCV	packed cell volume
ppm	parts per million
rt	room temperature
SDS-PAGE	sodium dodecyl sulfate polyacrylamide gel electrophoresis
TB	terrific broth
TMPBr	tetramethylphosphonium bromide

Supporting Figures and Tables

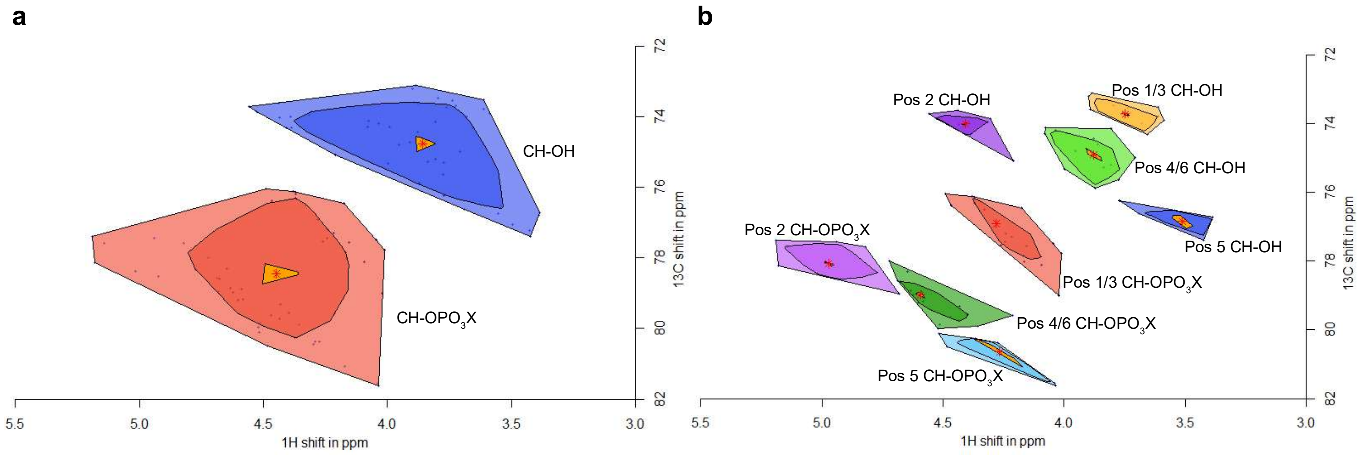

Figure S1: The bagplots illustrate the clustering depending on phosphororylation state (**a**) (blue: OH groups, red: phosphorylated groups) and position on the inositol ring (**b**). A fence factor of 6 was used to include all data points in the respective bags, the underlying data is the same as shown in Figure 2.

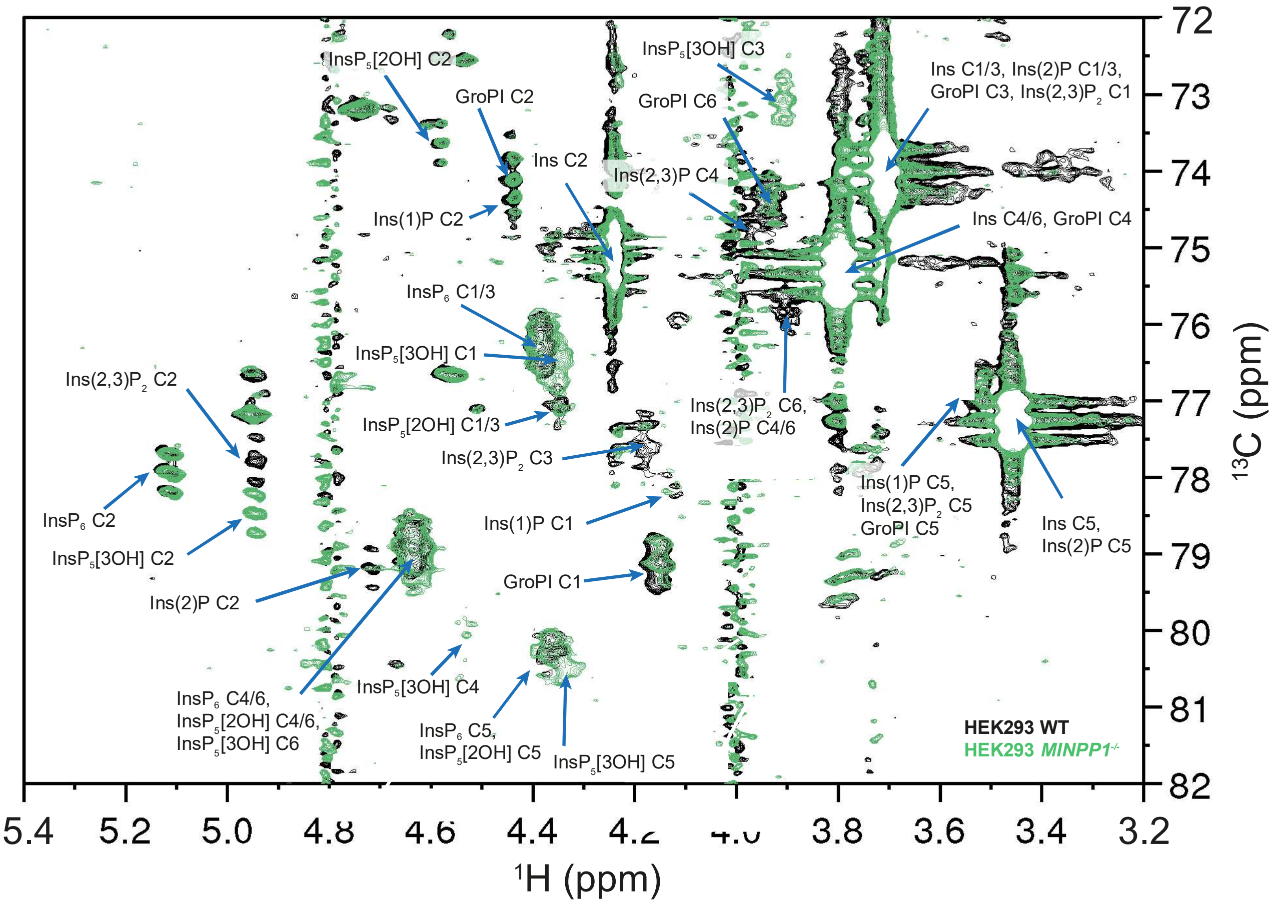

Figure S2: Complete annotation of [$^{13}C_6$]Ins-labeled HEK293 WT (black spectrum) and *MINPP1*$^{-/-}$ (green spectrum) metabolic extracts.

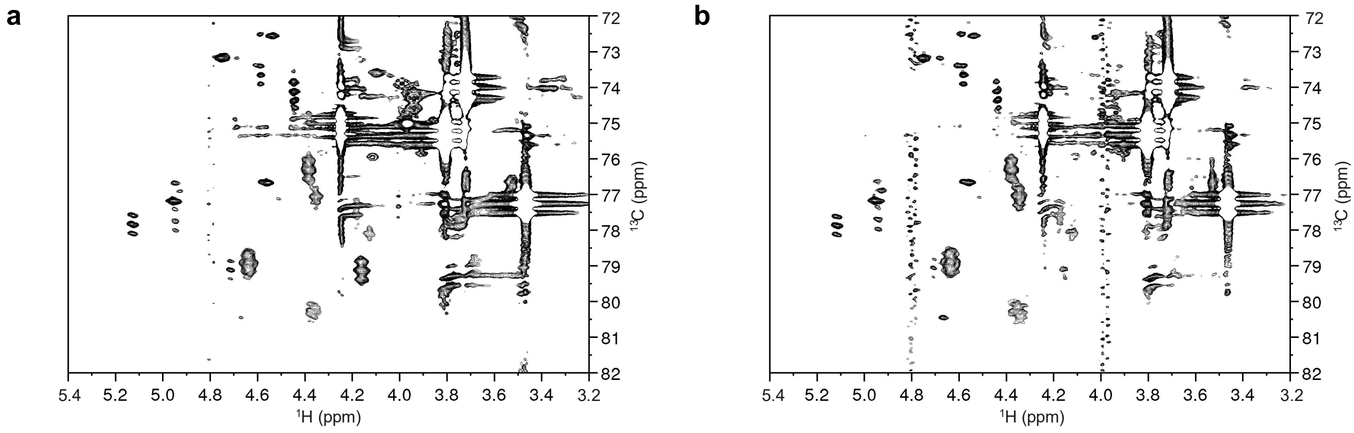

Figure S3: Additional NMR spectra of metabolic extracts from immortalized human wild-type cells. (**a**): HT29, (**b**): H1975. All labeled wild-type cells lines contain the same set of InsPs with varying concentrations.

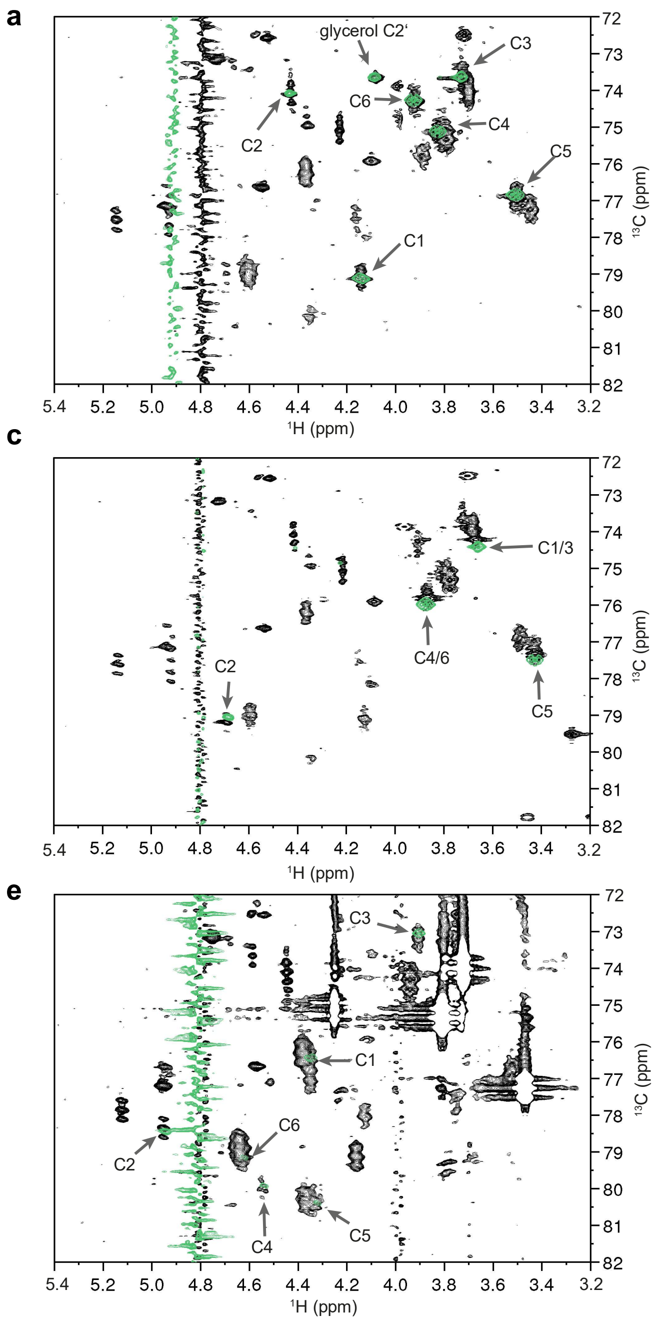

Figure S4: NMR spectra of [$^{13}C_6$]Ins-labeled H1Hela WT metabolic extracts spiked with InsP standards (black) overlayed with spectra of the same standard in saturated $KClO_4$ solution in D_2O, pH* = 6.0 (green). a: GroPI; b: Ins(1,2)P_2; c: Ins(2)P, d: Ins(1)P. e: NMR spectrum of [$^{13}C_6$]Ins-labeled HEK293 *MINPP1*$^{-/-}$ metabolic extract (black) overlayed with InsP_5[3OH] (green). The corresponding positions on the inositol ring are annotated with arrows. For Ins(1,2)P_2 the annotations for the spike-in standard are written in green while the annotation for the other enantiomer Ins(2,3)P_2, which is the species present in mammalian cells, are written in black. Note that in a and b the solvent signal is shifted between the extract and the InsP standards due to different sample temperatures during NMR measurement.

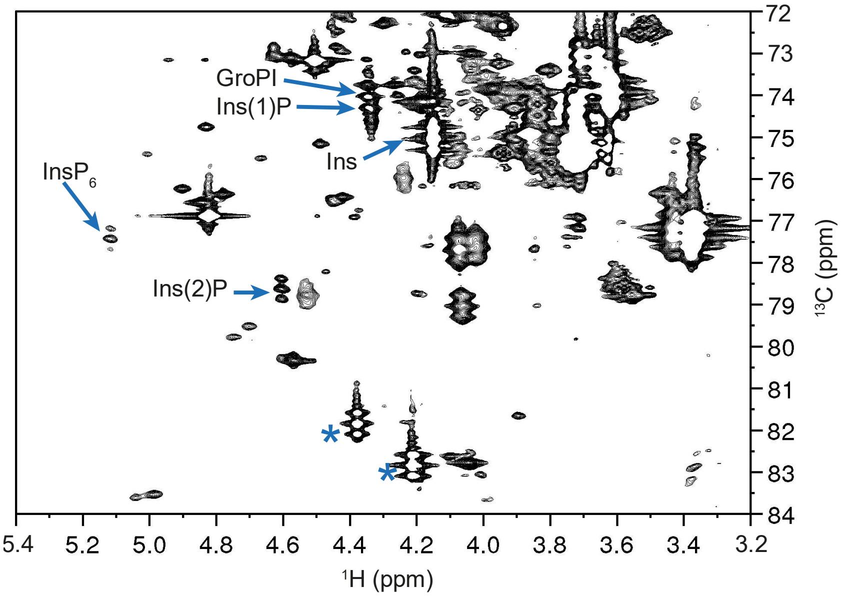

Figure S5: HMQC spectrum of metabolically [$^{13}C_6$]*myo*-inostiol-labeled *S. pombe*. The labeling protocol has already been published elsewhere.[1] While several InsPs were observed that overlap with mammalian InsP species ($InsP_6$, Ins(2)P, GroPI and Ins(1)P, annotation was limited to the 2-position for clarity), *S. pombe* extracts contain multiple high-intensity triplet signals that do not match any clusters established in Figure 2. Therefore, these *myo*-inositol-derived species likely do not represent *myo*-inositol phosphates. The exact identity of these metabolites will be addressed in future work.

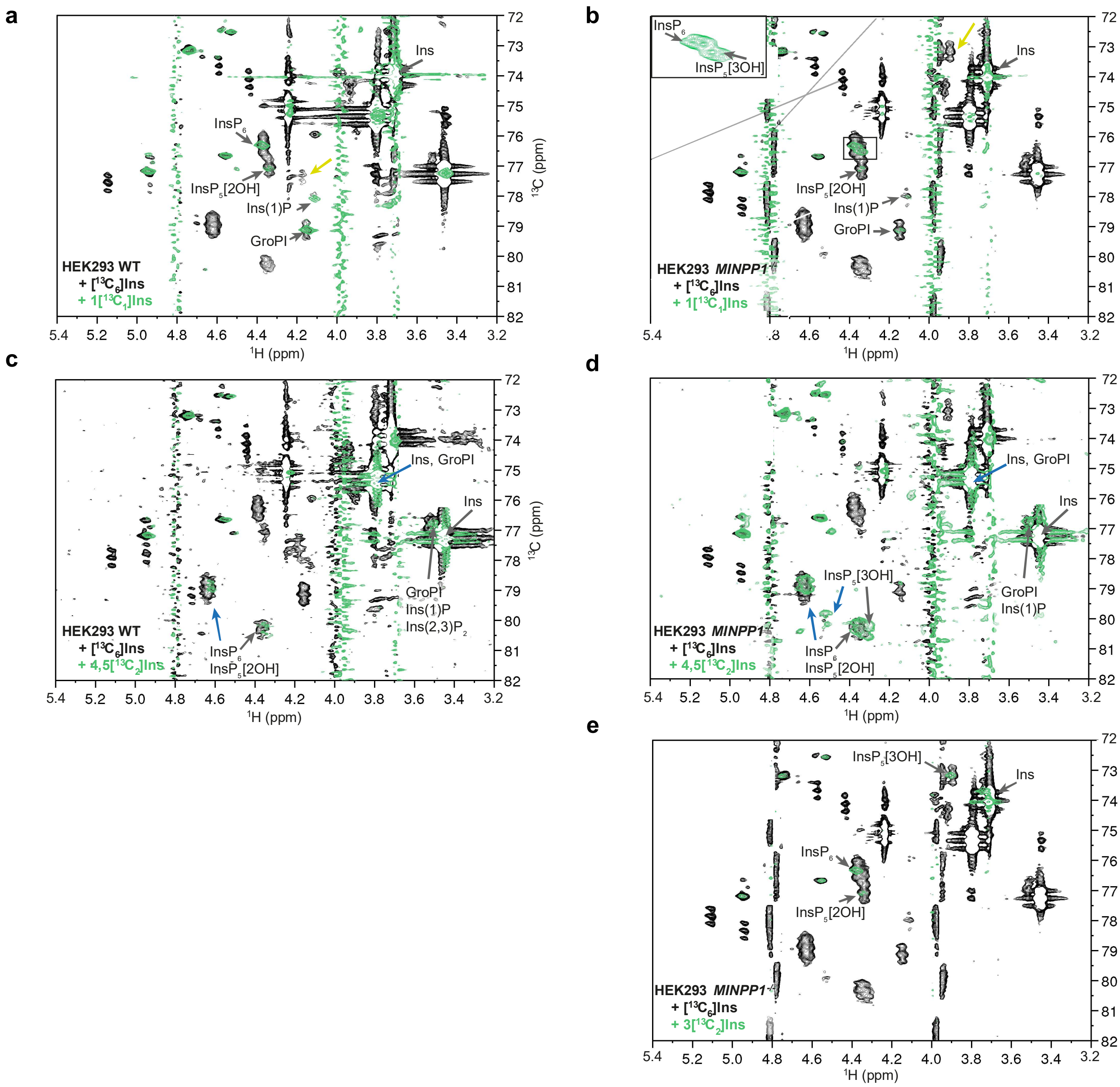

Figure S6: HMQC spectra of HEK cell lines which were metabolically labeled with asymmetrical isotopomers of *myo*-inositol (green) overlayed on the respective spectra of [$^{13}C_6$]*myo*-inositol-labeled cells (black). Annotations were limited to labeled positions. (a): 1[$^{13}C_1$]*myo*-inositol-labeled HEK293 WT cells (same data as in Fig. 3b). This labeling experiment illustrates that the signal for the phosphorylated position of Ins(1/3,2)P_2 (yellow arrow) is not labeled, thus excluding Ins(1,2)P_2 as a possible enantiomer. Note that non-labeled positions of *myo*-inositol are still visible due to the large relative abundance of *myo*-inositol in the extract. (b): 1[$^{13}C_1$]*myo*-inositol-labeled HEK293 *MINPP1*$^{-/-}$ cells. Here the signal for the 1-position of $InsP_5$[1/3OH] is clearly still phosphorylated (insert showing the magnified region), while the dephosphorylated 1/3-position (yellow arrow) is not labeled, indicating that the enantiomer present cannot be $InsP_5$[1OH], but must be $InsP_5$[3OH]. (c): 4,5[$^{13}C_2$]*myo*-inositol-labeled HEK293 WT cells. For 4,5[$^{13}C_2$]*myo*-inositol-labeled spectra the 4-positions are marked with a blue arrow and 5-positions with a black arrow. Note that the InsP signals now show a characteristic doublet pattern due to ^{13}C-^{13}C coupling. (d): 4,5[$^{13}C_2$]*myo*-inositol-labeled HEK293 WT *MINPP1*$^{-/-}$ cells. Here, the signal for the 4-position of $InsP_5$[3OH] is shifted away from the signals of the 4/6-positions of $InsP_6$/$InsP_5$[2OH], which is consistent with the observed shifts for the $InsP_5$[3OH] standard, but not $InsP_5$[1OH] (see also Figure S4e). (e):

3[$^{13}C_1$]*myo*-inositol-labeled HEK293 *MINPP1*$^{-/-}$ cells. The dephosphorylated position of $InsP_5$[1/3OH] is labeled, consistent with the enantiomer $InsP_5$[3OH].

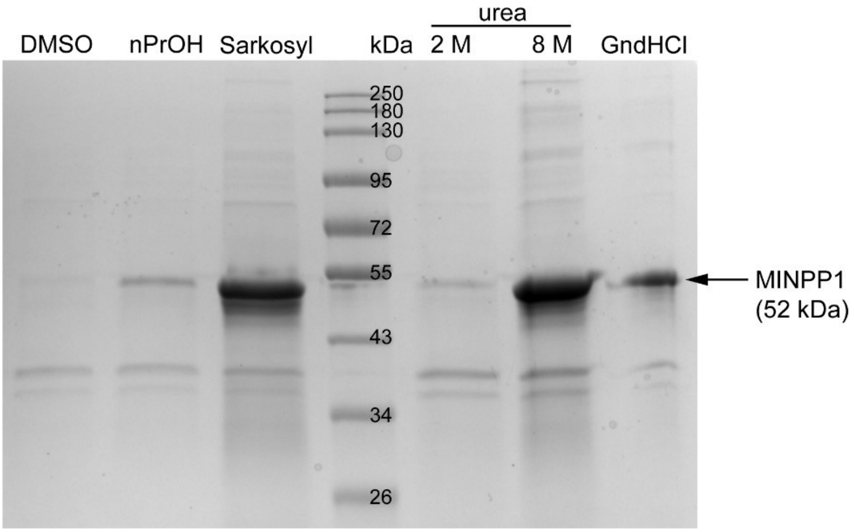

Figure S7: Testing resolubilization buffers for MINPP1 purification from inclusion bodies. MINPP1 was expressed according to the procedure described in the Experimental section. One part of the cell debris pellet obtained after lysis was washed only once with DI water, weighted, resuspended in little water and distributed into six 15 mL tubes (110 mg of wet pellet per tube). 4 mL of each resolubilization buffer (Experimental section under Cloning and production of MINPP1) were added to each tube, and incubated for 16 h at 4 °C on a reciprocal shaker. The tubes were centrifuged (30 min, 3000 g, 4 °C). 10 µL of supernatant were each diluted with 60 µL deionized water, 30 µL SDS running buffer, 40 µL Lämmli-buffer (incl. *β*-mercaptothanol) and all samples except for the guanidinium hydrochloride-based sample were boiled for 5 min at 90 °C. 30 µL of each sample were loaded on an SDS-PAGE gel, 150 V were applied until the loading marker completely ran into the gel. The wells were then flushed with SDS running buffer to remove excess guanidium hydrochloride to prevent gel distortions. Then the SDS-PAGE was continued (150 V, 45 min) and stained using colloidal Coomassie.

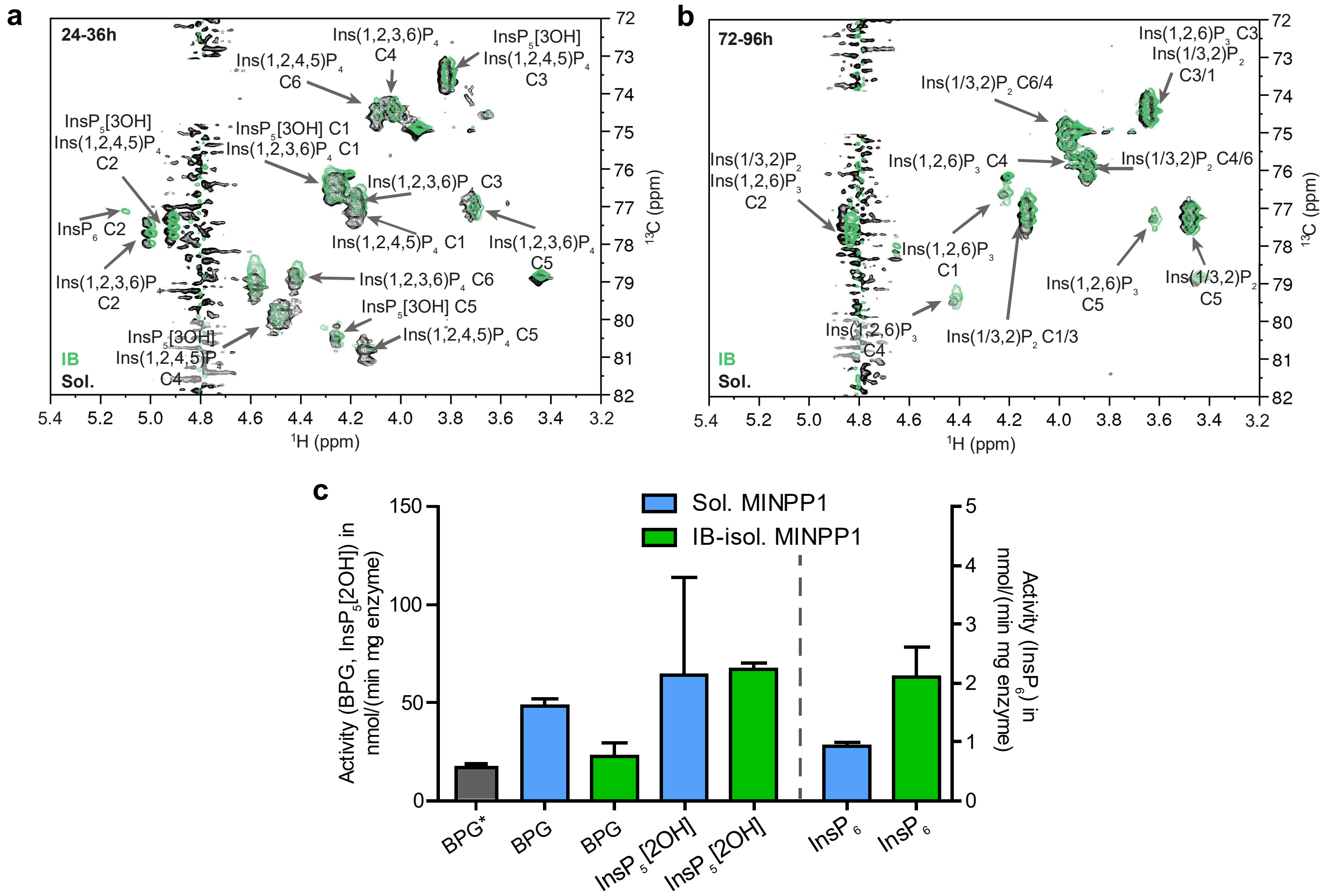

Figure S8: MINPP1 isolated from inclusion bodies (IB) exhibits similar properties to non-refolded MINPP1 obtained from the soluble fraction of *E. coli* lysate (Sol). (a) and (b): Sol. MINPP1 produces the same intermediates from [$^{13}C_6$]$InsP_6$ compared to IB MINPP1 (a: 24-36h, b: 72-96h). (c): Reaction rates of Sol. MINPP1 and IB MINPP1 against different substrates determined by Malachite green assay are similar. 2,3-bisphosphoglycerate (BPG), $InsP_5$[2OH] (left y-axis) and $InsP_6$ (right y-axis) were incubated with Sol. MINPP1 or IB MINPP1 as described in the Experimental section. Shown in grey is V_{max} determined by Cho *et al.*.[2]

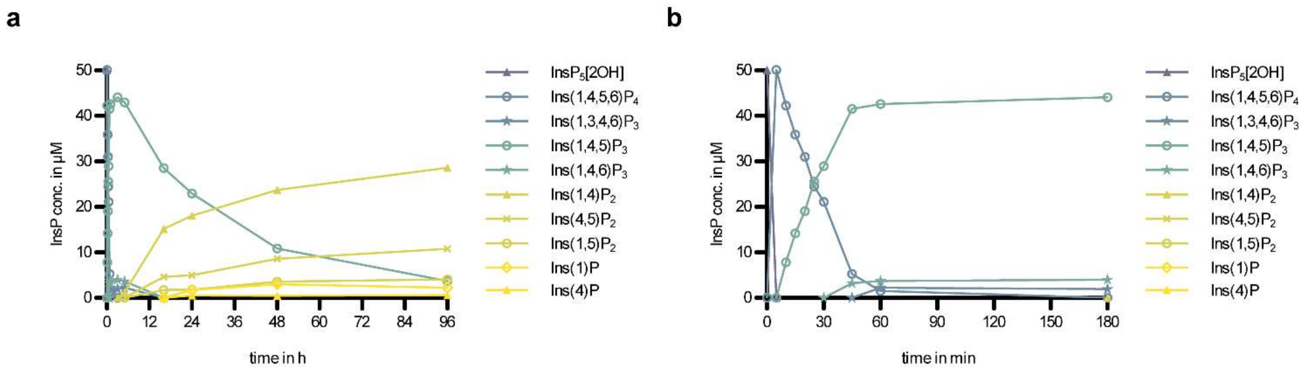

Figure S9 Progress curves of MINPP1 reaction with 50 µM $InsP_5$[2OH] (a and the first 180 min in b).

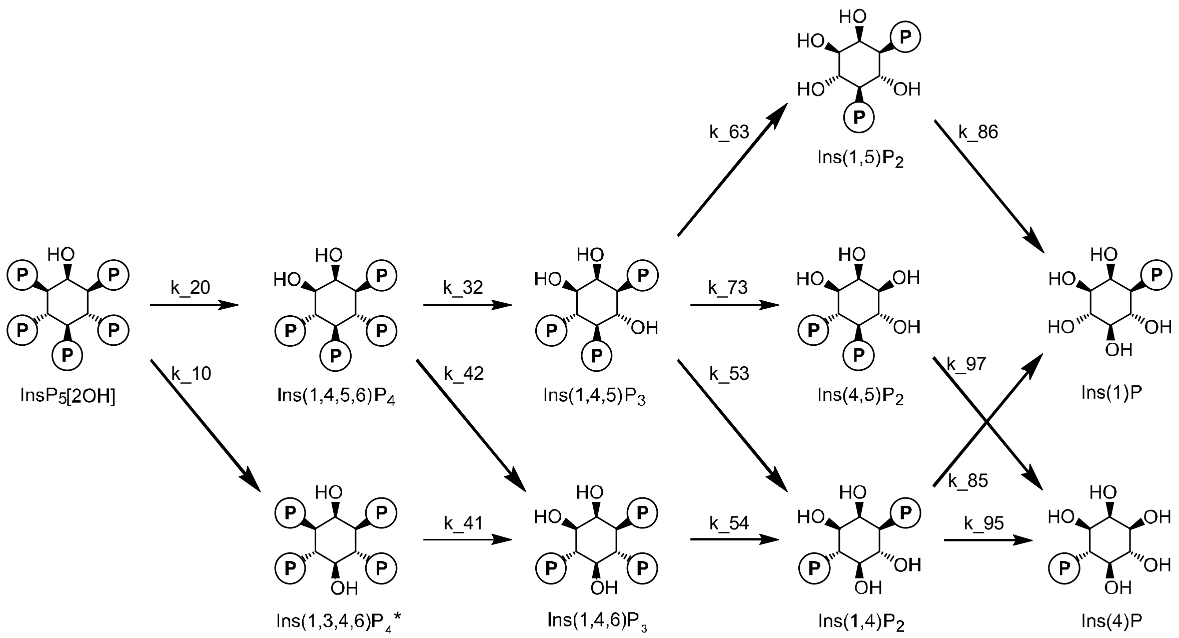

Figure S10: Complete MINPP1-mediated dephosphorylation pathway observed for $InsP_5$[2OH]

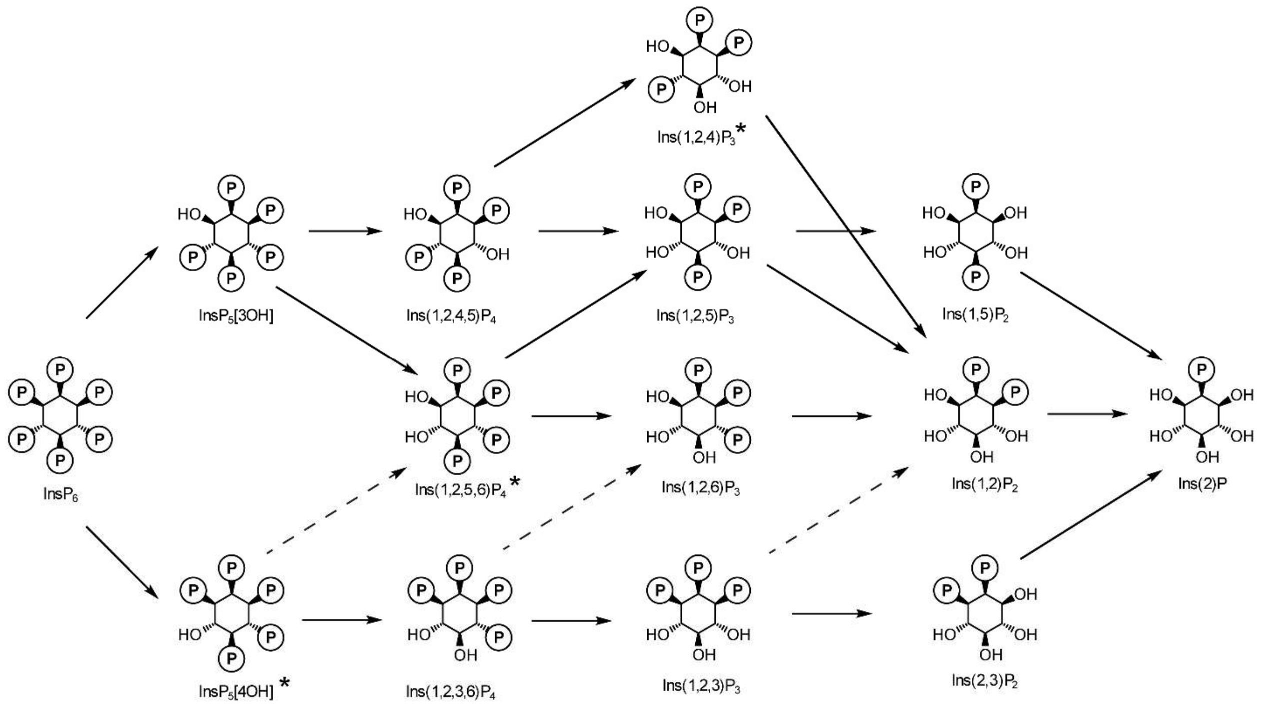

Figure S11: Complete MINPP1-mediated dephosphorylation pathway observed for $InsP_6$. The dashed arrows indicate theoretically possible paths which we assume are not relevant to the overall outcome. More investigation is needed to confirm this.

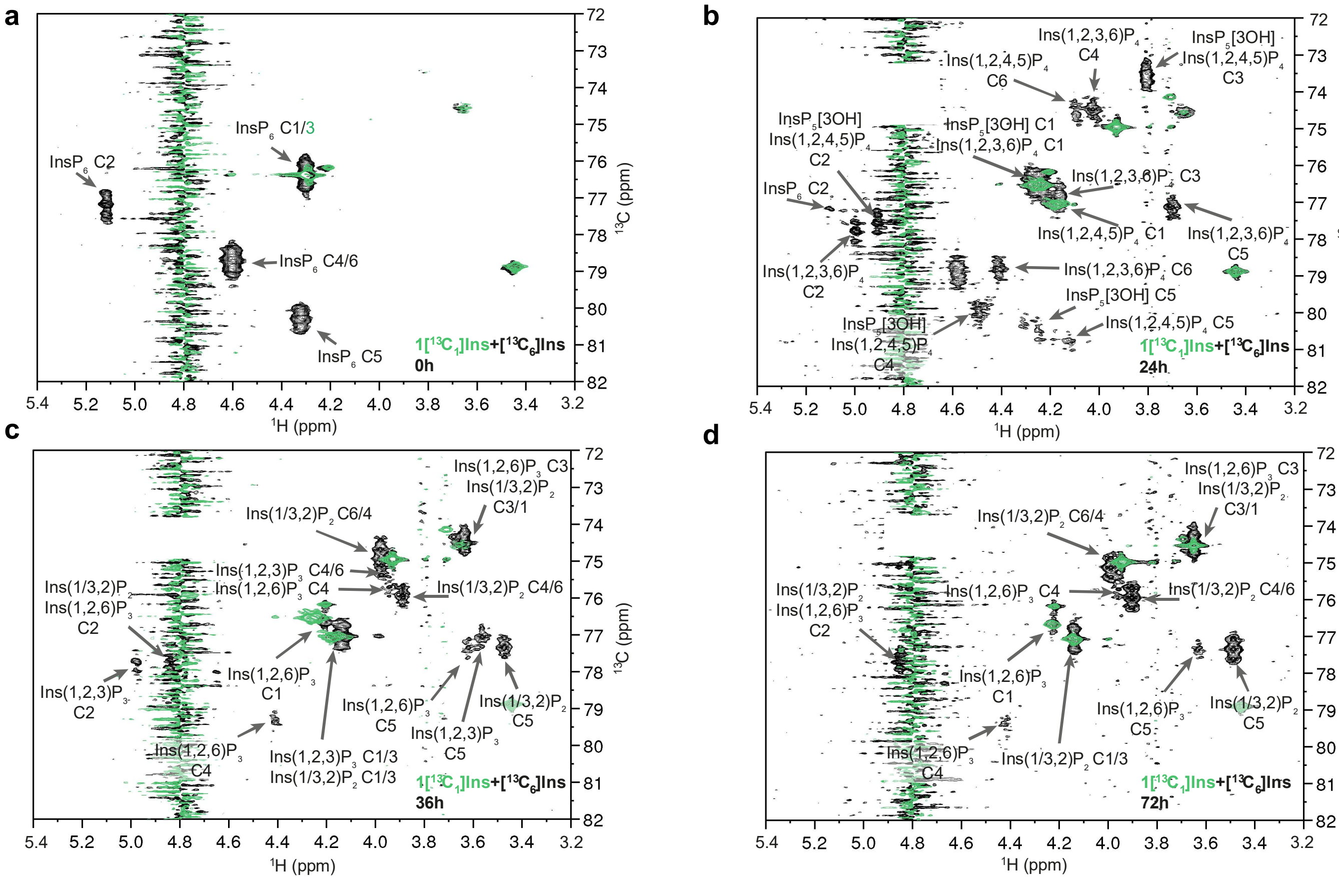

Figure S12: MINPP1 dephosphorylation of $1[^{13}C_1]InsP_6$. The spectra show reactions in which MINPP1 was incubated with either 175 µM $1[^{13}C_1]InsP_6$ (green) or a 1:1 mixture of $[^{13}C_6]Ins:1[^{13}C_1]Ins$ (black). (**a**): Control sample without enzyme. $InsP_6$ is clearly shown to be labeled at the 1-position. The other visible signals belong to buffer components. (**b**): Reaction mixture after 24 h of incubation. The 1-position is not dephosphorylated at this stage. Thus, the shown enantiomers are enantiopure. The 3-position of $Ins(1,2,3,6)P_4$ is slightly shifted upfield with regards to the ^{13}C-dimension, compared to the labeled 1-position of $Ins(1,2,4,5)P_4$. Also, the signals at ~75 and ~79 ppm (^{13}C dimension) are buffer components from the MINPP1 stock solution. (**c**): Reaction mixture after 36 h incubation. The 1-position of $Ins(1,2,6)P_3$ and 3/1-position of $Ins(1/3,2)P_2$ overlap with the buffer component at ~75 ppm which seems to increase in intensity. The labeling of the 1-position appearing in both the region for phosphorylated and the region for dephosphorylated positions indicate that a mixture of $Ins(2,3)P_2$ and $Ins(1,2)P_2$ is formed. (**d**): Reaction mixture after 72 h of incubation. The dephosphorylated 1-position of $Ins(2,3)P_2$ is now evident while the 1-position of $Ins(1,2)P_2$ is still phosphorylated, indicating that a mix of both enantiomers has been formed, despite the enantio-specific nature of the previous dephosphorylation steps in (b). A rough integration of all labeled 1-position signals (green spectrum) resulted in a near 1:1 ratio between the dephosphorylated 1-position of $Ins(2,3)P_2$ and the combined phosphorylated 1-position of $Ins(1,2)P_2$ and $Ins(1,2,6)P_3$. This high ratio suggests that MINPP1 likely converts $Ins(1,2,3)P_2$ exclusively into $Ins(2,3)P_2$, while all intermediates downstream of $InsP_5[3OH]$ must result in the other enantiomer $Ins(1,2)P_2$.

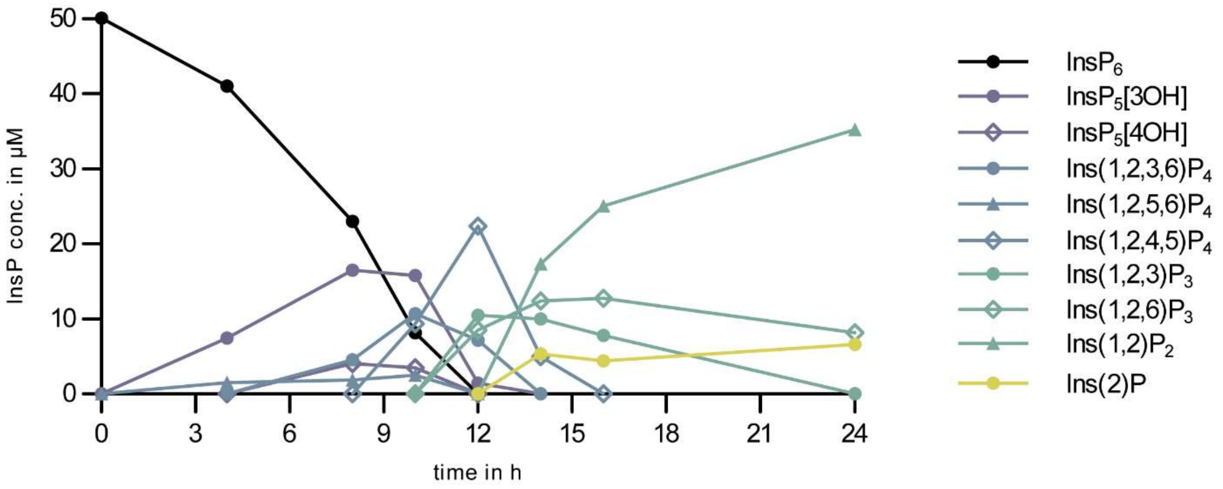

Figure S13: Due to substrate inhibition the MINPP1 progress curves for $InsP_6$ show different kinetics with respect to the dephosphorylation intermediates at lower (50 µM) initial concentrations of $InsP_6$, compare also with Figure 5c (175 µM initial concentration). Representative of 3 replicates.

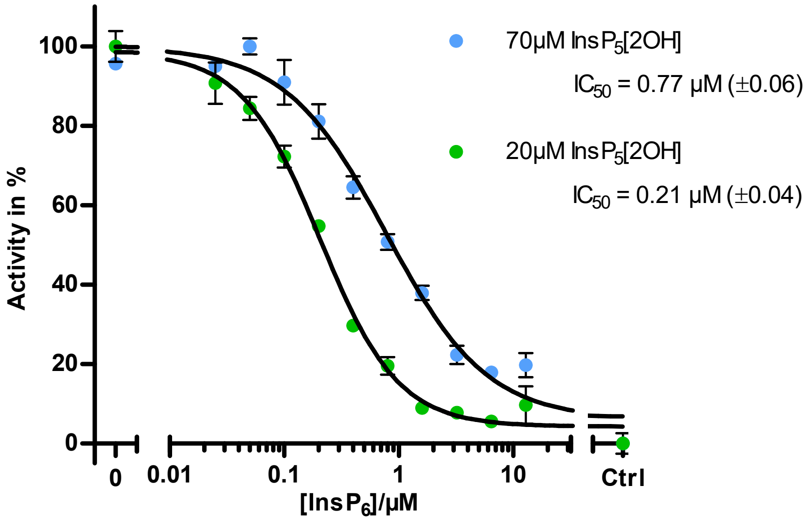

Figure S14: $InsP_6$ inhibits the MINPP1-mediated dephosphorylation of different substrate concentrations of $InsP_5$[2OH] with changing IC_{50}-values in agreement with the Cheng-Prusoff equation. Either 70 or 20 µM $InsP_5$[2OH] were incubated with 0.5 µM MINPP1 and different amounts of $InsP_6$ (two-fold dilution series ranging from 12.8 – 0.025 µM final $InsP_6$ concentration) and phosphate release was determined using a Malachite green-assay kit after 24 min reaction time (20 µM) or 1 h (70 µM). IC_{50}-values are reported with standard error of $\log_{10}IC_{50}$ in brackets. Starting from the determined IC_{50} = 1.97 (±0.02) at 175 µM substrate (see Fig 6c), the expected IC_{50}-values according to the Cheng-Prusoff equation assuming competitive inhibition are 0.77-0.82 µM for 70 µM substrate (found: 0.77 µM (±0.06)) and 0.22-0.23 µM for 20 µM substrate (found: 0.21(±0.04)). With starting concentrations far above the Michaelis-Menten constant for $InsP_5$[2OH] (40 nM), un- and non-competitive inhibition would show a substrate-concentration independent IC_{50}.[3]

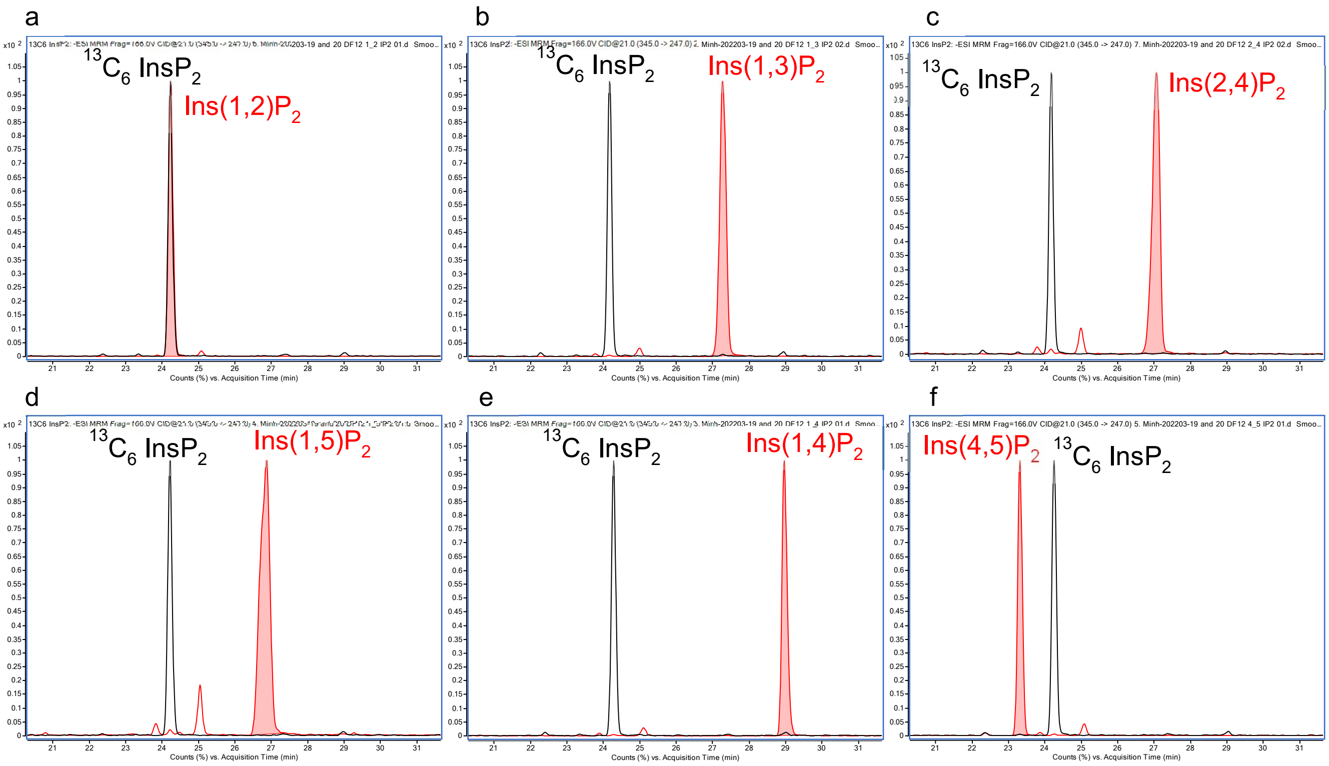

Figure S15: Confirmation of the identity of $Ins(1/3,2)P_2$ *via* CE-MS. The metabolic extract of $[^{13}C_6]$Ins metabolically-labeled HEK293 WT cells were spiked with commercial standards of different $InsP_2$ isomers and analyzed *via* CE-MS. Depicted are the extracted ion chromatograms corresponding to the masses of the intracellularly synthesized $[^{13}C_6]InsP_2$ (black) and the non-labeled $InsP_2$ standards. Only $Ins(1,2)P_2$ coelutes with the $[^{13}C_6]InsP_2$ signal in question (a) while all other tested $InsP_2$ standards (b: $Ins(1,3)P_2$, c: $Ins(2,4)P_2$, d: $Ins(1,5)P_2$, e: $Ins(1,4)P_2$, f: $Ins(4,5)P_2$) do not.

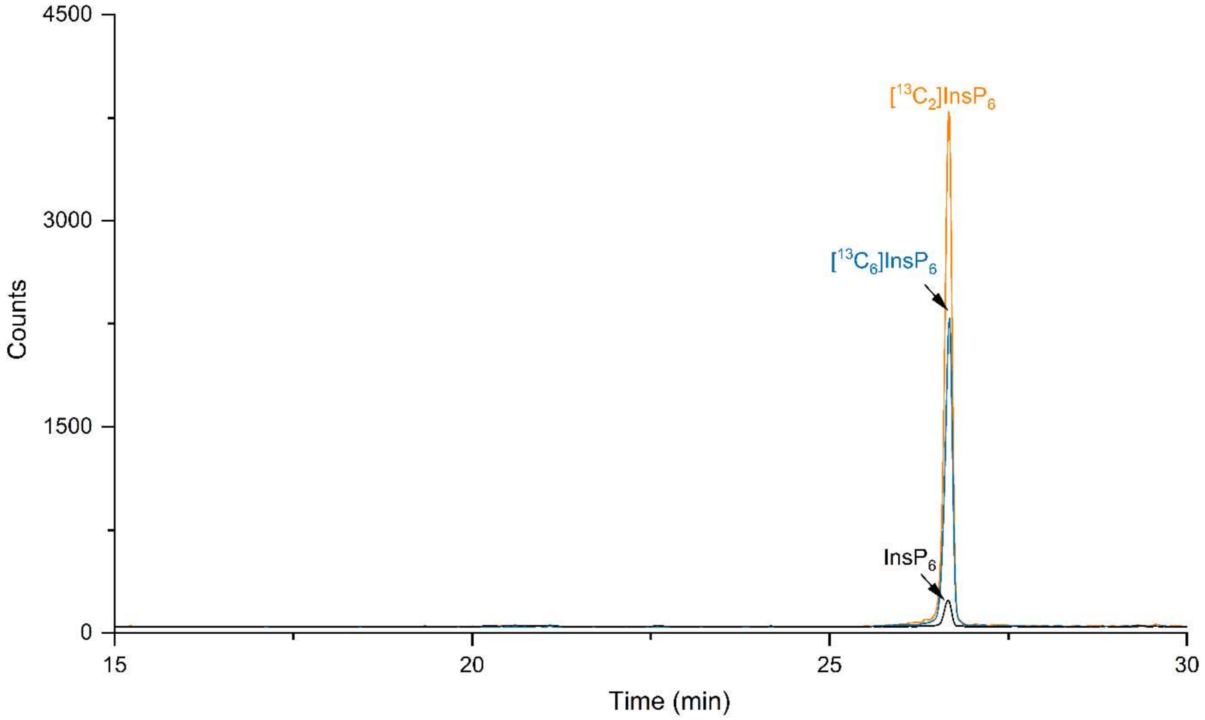

Figure S16: Example EICs (extracted ion chromatograms) of [13$C_{6/2/0}$]InsP_6 in a HEK293 WT cells which were metabolically labeled with [$^{13}C_6$]*myo*-inositol to equilibrium and then with 4,5[$^{13}C_2$] *myo*-inositol for 48h. For the metabolic flux analysis (Figures 7b, 7c) the integrals of the respective isotopomer peaks (blue/ orange) were used for relative quantification. The InsP pools contained a constant ~3% of non-labeled InsPs (black) due to glucose-6-phosphate-dependent neogenesis of *myo*-inositol.

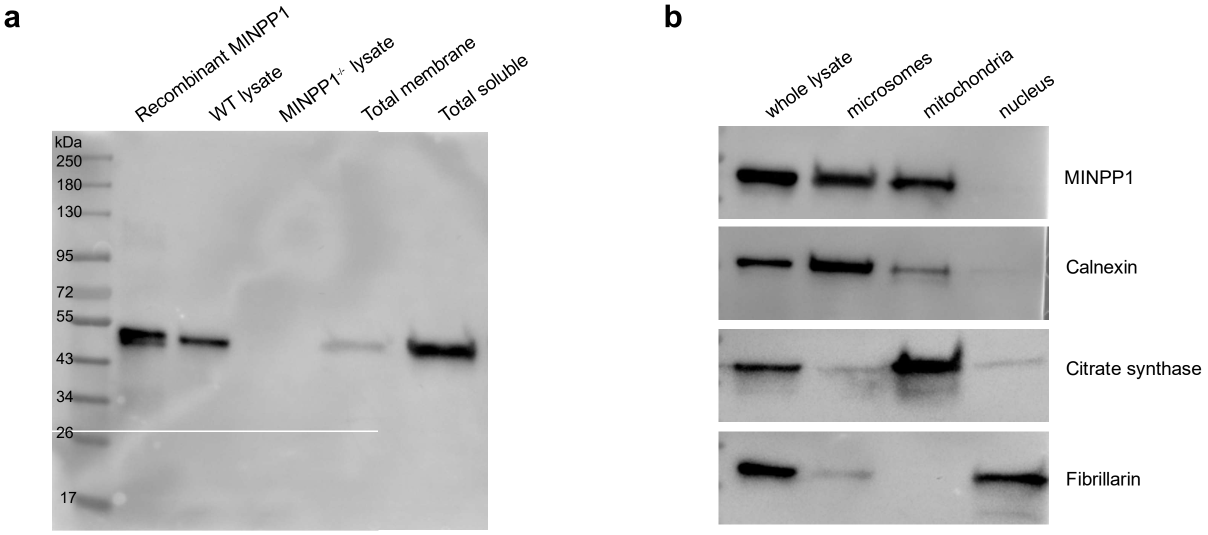

Figure S17: MINPP1 Western blots of HEK293 WT and *MINPP1*$^{-/-}$ cells and subcellular fractions. (a): MINPP1 is present in HEK293 WT cell lysates but expectedly not in MINPP1$^{-/-}$ HEK lysates and is predominantly found in the soluble fraction. (b): MINPP1 is not found in the nucleus but in ER (microsomes) and mitochondria. Calnexin was used as an ER marker, citrate synthase as a mitochondrial marker and fibrillarin as a nuclear marker.

Experimental section

Safety statement

No unexpected or unusually high safety hazards were encountered.

General Information

Chemicals were obtained from Sigma Aldrich, VWR, Roth, TCI, Thermo Scientific or Roche and used without further purification unless stated otherwise.

InsP standards were purchased as sodium, potassium, ammonium or cyclohexylammonium salts from SiChem (Ins(3,4,5,6)P_4, Ins(1,4,5,6)P_4, Ins(1,4,5)P_3, Ins(1,3,4)P_3, InsP_5[3OH], InsP_5[1OH]), Cayman chemical (Ins(2,3,5)P_3, Ins(1,2)P_2), Echelon Bioscience (Ins(1,4)P_2, Ins(1,2,6)P_3, GroPI), Biomol (Ins(1,5)P_2, Ins(1,4,6)P_2) or Sigma-Aldrich (Ins(1)P, Ins(2)P) or synthesized in-lab ([$^{13}C_6$]InsP_6, [$^{13}C_6$]InsP_5[2OH], [$^{13}C_6$]1PP-InsP_5, [$^{13}C_6$]5PP-InsP_5, [$^{13}C_6$]1,5(PP)$_2$InsP_4) as described previously.[4] Non-labeled InsPs were dissolved in a saturated $KClO_4$ solution in D_2O (pH* 6.0) to mimick the conditions of the metabolic extracts. Non-labeled standards were dissolved in the smallest volume possible for NMR measurements (min. 500 µL). All samples were adjusted to pH* 6.0 if necessary using DCl and NaOD solutions in D_2O (all deuterated solutions obtained from Eurisotop).

For NMR-based quantification purposes standards (TMPBr (Sigma, 288268) or phosphonoacetic acid (TraceCert ^{31}P-NMR standard, Supelco, 79251), respectively) were dissolved/ diluted in dry D_2O (Eurisotop D215T) and aliquots are frozen until use.

NMR data acquisition and processing

For NMR measurements and NMR data analysis TopSpin 3.5 was used. Measurements were conducted on a Bruker AV-III spectrometer (Bruker Biospin, Rheinstetten, Germany) operating at 600 MHz for ^{1}H and 151 MHz for ^{13}C nuclei equipped with a cryo-QCI probe. The pulse sequence for BIRD-{^{1}H,^{13}C}HMQC is based on the hmqcbiph pulse program from Bruker. Measurement parameters are adapted depending on sample composition. Typically, metabolic extracts were recorded with TD(^{13}C) = 1024, 140 scans, spectral width (^{13}C) limited to 40 – 100 ppm. Typically, samples from *in vitro* experiments were recorded with TD (^{13}C) = 512, 64 scans, spectral width (^{13}C) limited to 50 – 90 ppm. All samples were recorded at 310 K.

BIRD-{^{1}H,^{13}C}HMQC-NMR spectra were processed without digital water suppression with manual phasing and automatic baseline correction.

Quantification of NMR data were conducted as follows: For metabolic extracts InsPs were quantified against a known concentration of tetramethylphosphonium bromide (TMPBr). A standard curve for InsP_6 and InsP_5[2OH] against TMPBr was recorded earlier [5]. For other InsP species the standard curve for InsP_6 was used as an approximation as there are no fully ^{13}C-labeled standards available. For the samples from the *in vitro* dephosphorylation of Ins$P_{6/5}$ by MINPP1 the InsP signals were quantified relatively to each other and normalized to a total InsP concentration matching the initial substrate concentration. As the signals of the 2-positions are the sharpest and best resolved (due to the reduced coupling to the neighbouring CH groups), the 2-position signals were used for quantification. In the cases where the 2-position signals of two InsPs species are not baseline-separated, the signals were integrated together and split by the ratio of the 5-position signal integrals.

CE-MS measurement

CE-ESI-MS has been found to be an efficient platform for the analysis of inositol polyphosphate.[6] A CE-ESI-QQQ setup is used for this study, which consists of an Agilent 7100 CE, a triple quadrupole tandem mass spectrometry Agilent 6495c, connected to an Agilent Jet Stream (AJS) electrospray ionization (ESI) source. A commercial CE-MS sheath liquid coaxial interface was used, with an isocratic LC pump constantly delivering the sheath-liquid (*via* a splitter set with a ratio of 1:100). All experiments were performed on a bare fused silica capillary with a length of 100 cm (50 µm internal diameter and 365 µm outer diameter). 35 mM ammonium acetate titrated by ammonia solution to pH 9.7 was employed as

background eletrolyte (BGE). Samples were injected by applying 100 mbar pressure for 15 s, corresponding to 1.5% of the total capillary volume (30 nL).

The sheath liquid is a mixture of water-isopropanol (1/1, v/v) and with a constant flow of 10 µL/min. The MS source parameters settings were as follows: nebulizer pressure was set to 8 psi, gas temperature was 150 °C with a flow of 11 L/min, sheath gas temperature was 175 °C and with a flow of 8 L/min, capillary voltage was -2000 V with nozzle voltage 2000 V. Negative high-pressure RF and low-pressure RF (Ion Funnel parameters) were 70 V and 40 V, respectively. Mass spectrometer parameters for MRM transitions are shown below.

**Compound Name**	**Precursor Ion**	**Product Ion**	**dwell**	**Frag (V)**	**CE (V)**	**Cell Acc (V)**	**Polarity**
$[^{13}C_6]InsP_6$	331.9	486.9	60	166	13	4	Negative
$[^{13}C_2]InsP_6$	329.9	482.9	60	166	13	4	Negative
$[^{12}C_6]InsP_6$	328.9	480.9	60	166	13	4	Negative
$[^{13}C_6]InsP_5$	292	504.9	60	166	9	3	Negative
$[^{13}C_2]InsP_5$	290	500.9	60	166	9	3	Negative
$[^{12}C_6]InsP_5$	289	498.9	60	166	9	3	Negative
$[^{13}C_6]InsP_4$	252	424.9	60	166	5	1	Negative
$[^{13}C_2]InsP_4$	250	420.9	60	166	5	1	Negative
$[^{12}C_6]InsP_4$	249	418.9	60	166	5	1	Negative
$[^{13}C_6]InsP_2$	345	247	60	166	21	4	Negative
$[^{13}C_2]InsP_2$	341	243	60	166	21	4	Negative
$[^{12}C_6]InsP_2$	339	241	60	166	21	4	Negative

Data handling

For plotting and other analyses Microsoft Excel, OriginPro 2016 and GraphPad Prism 5 were used. Bagplots were created in R (version 4.1.2) with the aplpack package (version 1.3.5).

For details of the kinetic modelling of MINPP1 see separate SI file “Supporting Information: Numerical Analysis”.

Synthesis of ^{13}C-labeled Ins and InsPs

The synthesis of ^{13}C-labeled *myo*-inositol and its derivatization to InsPs were carried out based on published procedures for $[^{13}C_6]$Ins with slight improvements of the protocol as described below.[5]

Chemoenzymatic synthesis of *myo*-inositol isotopomers

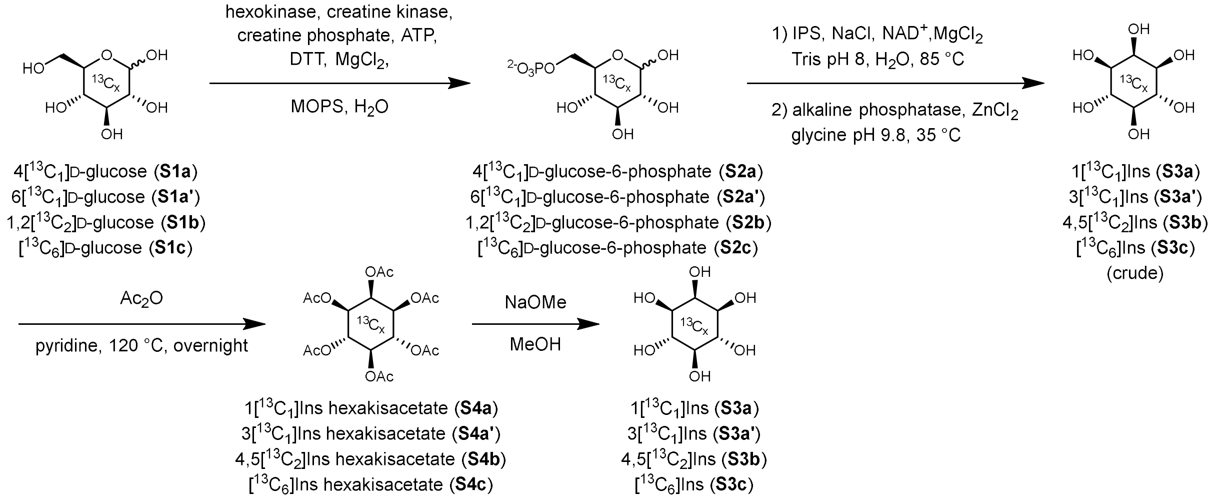

Ins isotopomers were synthesized chemoenzymatically from the respective D-glucose isotopomer: 1[$^{13}C_1$]Ins (**S3a**) was synthesized from 4[$^{13}C_1$]D-glucose (**S1a**), 3[$^{13}C_1$]Ins (**S3a'**) was synthesized from 6[$^{13}C_1$]D-glucose (**S1a'**), 4,5[$^{13}C_2$]Ins (**S3b**) was synthesized by starting from 1,2[$^{13}C_2$]D-glucose (**S1b**), and [$^{13}C_6$]Ins (**S3c**) from [$^{13}C_6$]D-glucose (**S1c**). ^{13}C-labeled material was obtained from Eurisotop/ Cambridge Isotope Labs. Generally, we observed improved yields with higher synthesis scale with 1 to 3 g glucose as starting material yielding up to 55% Ins. However, the asymmetric isotopomer **S3a** was synthesized only on a 500 mg scale.

Briefly, **S1a/a'/b/c** is first converted enzymatically to the respective D-glucose-6-phosphate (**S2a/a'/b/c**) with hexokinase and crudely purified *via* an anion exchange hand column. The subsequent lyophilization step of the eluate in the original procedure can be replaced by concentrating using a rotavap without reduction of yield while saving time. The resulting product/salt mixture is then converted to inositol-3-monophosphate (Ins(3)P) through the action of inositol monophosphate synthase (IPS), which is monitored *via* NMR. We recommend preparing recombinantly expressed IPS as closely to the protocol in [5] as possible to ensure sufficient activity of the IPS (esp. induction at high OD_{600} and purification *via* heat-treatment); prolonged reaction times causes the NAD^+ cofactor to degrade, inhibiting IPS activity even after resupplementing more IPS and NAD^+. Subsequently, Ins(3)P is dephosphorylated to Ins (**S3a/a'/b/c**) by alkaline phosphatase. The reaction progress is also monitored *via* NMR. The ion exchange treatment in the original procedure can be skipped upon complete conversion and the aqueous solution can be reduced on a rotavap instead, yielding a crude brown solid. The Ins is then purified through chemical derivatization by acetylation to *myo*-inositol hexakisacetate (**S4a/a'/b/c**), purification *via* extraction and column chromatography on silica gel (~500 mL silica gel for a 3 g synthesis scale), followed by deacetylation and precipitation in acetonitrile (the precipitation is repeated twice if necessary) to afford the desired *myo*-inositol isotopomer **S3a/a'/b/c** in pure form following the published protocol.

<u>1[$^{13}C_1$]Ins (S3a)</u>: yield: 122 mg (starting from 500 mg **S1a**, 24%)

^{1}H NMR (600 MHz, D_2O) δ[ppm]: 3.99 (s, 1H, 2-position), 3.56 (ps-q, J = 9.8 Hz, 2.5H, 4/6-position and 1-position), 3.46 (d, J = 9.9 Hz, 1H, 3-position), 3.34 (d, J = 11.4 Hz, 0.5H, 1-position), 3.21 (t, J = 9.5 Hz, 1H, 5-position). Please note that the 1-position is coupling with ^{13}C with a coupling constant of $^1J_{CH}$ = 143.4 Hz.

^{13}**C NMR** (151 MHz, D_2O) δ[ppm]: 77.09 (d, *J* = 6.7 Hz, 5-position), 75.17 (d, *J* = 33.4 Hz, 6-position), 75.14 (s, 4-position), 74.91 (d, *J* = 32.4 Hz, 2-position), 73.88 (large s, satellite d, J = 39.0 Hz, 1- and 3-position).

HRMS *m/z*: $[M – H]^-$ calcd. for $^{13}C_1{}^{12}C_5H_{11}O_6$ 180.0595; found 180.0593.

3[^{13}C$_1$]Ins (S3a'): yield: 563 mg (starting from 1000 mg **S1a**, 56%)

^{1}**H NMR** (600 MHz, D_2O) δ[ppm]: 4.09 (dt, J = 5.3, 2.9 Hz, 1H, 2-position), 3.66 (m, 2.5H, 4/6-position and 3-position), 3.56 (dd, J = 10.1, 3.0 Hz, 1H, 1-position), 3.44 (dd, J = 9.9, 2.9 Hz, 0.5H, 3-position), 3.31 (t, J = 8.9 Hz, 1H, 5-position). Please note that the 3-position is coupling with ^{13}C with a coupling constant of $^1J_{CH}$ ≈ 140 Hz.

^{13}**C NMR** (151 MHz, D_2O) δ[ppm]: 77.12 (d, *J* = 6.8 Hz, 5-position), 75.20 (d, *J* = 33.9 Hz, 4-position), 75.17 (s, 6-position), 74.94 (d, *J* = 32.8 Hz, 2-position), 73.9 (large s, satellite d, J = 39.1 Hz, 3- and 1-position).

HRMS *m/z*: $[M – H]^-$ calcd. for $^{13}C_1{}^{12}C_5H_{11}O_6$ 180.0595; found 180.0593.

4,5[^{13}C$_2$]Ins (S3b): yield: 450 mg (starting from 1 g **S1b**, 45 %)

^{1}**H NMR** (600 MHz, D_2O) δ[ppm]: 4.19 (t, *J* = 3 Hz, 1H, 2-position), 3.75 (tdd, J = 144.3, 9.9, 4.2 Hz, 1, 4-position) 3.75 (td, *J* = 9.7, 4.7 Hz, 1H, 6-postition), 3.66 (d, *J* = 9.8 Hz, 2H, 1/3-position), 3.40 (tdd, *J* = 140.7, 9.3, 4.1 Hz, 1H, 5-position).

^{13}**C NMR** (151 MHz, D_2O) δ[ppm]: 77.23 (d, *J* = 38.8 Hz, 5-position), 75.28 (d, *J* = 38.9 Hz, 4+6position), 75.05 (2-position), 74.02 (d, *J* = 6.8 Hz, 1-position), 74.00 (dd, *J* = 39.5, 7.1 Hz, 3-position).

HRMS *m/z*: $[M – H]^-$ calcd. for $^{13}C_2{}^{12}C_4H_{11}O_6$ 181.0628; found 181.0627.

[^{13}C$_6$]Ins (S3c): yield: up to 1.55 g (starting from 3 g **S1c**, 52%)

Analytical data for [^{13}C$_6$]Ins were published previously. [5]

Synthesis of 1[^{13}C$_1$]InsP$_6$

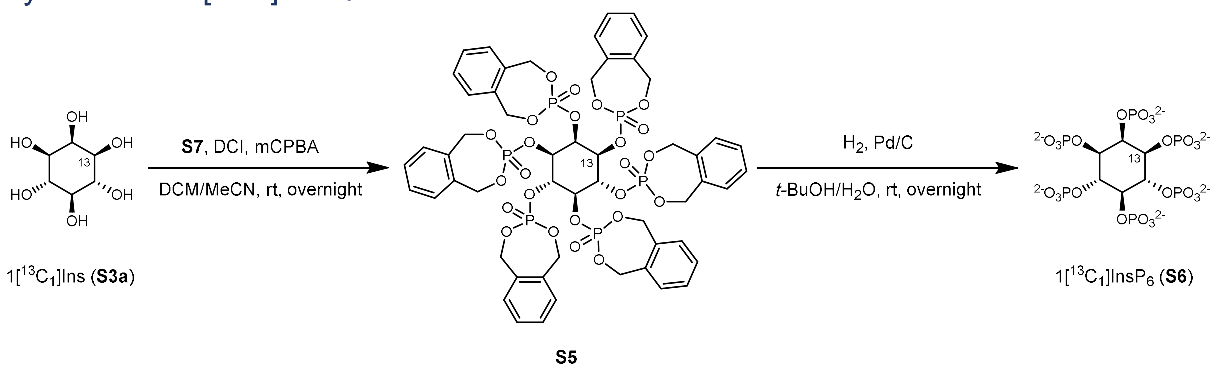

Synthesis of 1[^{13}C$_1$]InsP$_6$ (**S6**) was carried out following published procedures [5] with slight modifications:

1[$^{13}C_1$]Ins (30 mg, 0.17 mmol) is resuspended together with commercial *o-xylylene N,N-diethylphosphoramidite (**S7**)* (Sigma Aldrich, 360 mg, 1.5 mmol) and a stirring bar in anhydrous acetonitrile under nitrogen atmosphere. To reduce water content further, the suspension is reduced and evaporated under high vacuum for an hour. The dried mixture is then resuspended in 5.5 mL of 1:1 anhydrous dichloromethane:acetonitrile and sonicated briefly. The mixture is cooled to 0 °C using an acetone bath to which dry ice was added in a controlled manner. 4,5-DCI (254 mg, 2.15 mmol) was added and the gas phase was exchanged three times against nitrogen. The reaction is allowed to warm to room temperature and stirring is continued overnight under nitrogen/argon atmosphere. The subsequent workup is identical as described preciously [5] yielding **S5** in 68% yield (144 mg, 0.116 mmol) with slight impurities.

S5:

^{1}H NMR (600 MHz, $CDCl_3$) δ[ppm]: 7.40 – 7.31 (m, 20H), 7.27 – 7.25 (m, 4H, overlaps with solvent signal signal), 5.75 (dd, *J* = 13.8, 9.3 Hz, 2H), 5.65 (dt, *J* = 13.0, 8.0 Hz, 3H), 5.59 – 5.51 (m, 6H), 5.39 (dd, *J* = 13.8, 12.3 Hz, 2H), 5.30 – 4.93 (m, 18H).

^{13}C NMR (151 MHz, $CDCl_3$) δ[ppm:] 138.54, 138.51, 138.35, 138.16, 137.29, 132.34, 132.21, 132.08, 132.07, 132.04, 132.00, 131.83, 131.75, 80.01, 79.80, 79.59, 76.59, 76.56, 76.54, 72.46, 72.40, 72.29, 72.24, 72.19, 72.14, 72.11, 72.06.

^{31}P NMR (243 MHz, $CDCl_3$) δ[ppm]: -2.81 (d, *J* = 2.9 Hz, 1P), -3.37 (d, *J* = 3.0 Hz, 2P), -4.37 (s, 1P), -4.53 (s, 1P).

HRMS *m/z*: $[M + H]^+$ calcd. for $^{13}C_1{}^{12}C_{53}H_{55}O_{24}P_6$ 1274.1537; found 1274.1526.

144 mg (**S5**, 0.116 mmol, 1 eq.) was dissolved in 28 mL *t*-BuOH and Milli-Q® water 6:1, and 250 mg of palladium black (10% Pd/C) was added. The suspension was stirred overnight under hydrogen atmosphere. Upon depletion of starting material (according to LC-MS analysis) 2 ml Milli-Q® water was added to adjust the solvent to a ratio of 4:1 *t*-BuOH:Milli-Q® and stirring under hydrogen atmosphere was continued overnight. The catalyst was removed by centrifuging the suspension in 50 ml centrifugal tubes at 3000 g for 15 min and the supernatant was passed through a PTFE syringe filter (0.45 µm). The catalyst pellet is washed once with 5 ml Milli-Q® water, centrifuged and the supernatant is again filtered and the filtrates are united and tBuOH is removed on the rotavap before the aqueous solution is lyophilized. The resulting white solid is redissolved in 200 ml water and magnesium chloride solution is added to a final concentration of 26 mM (49 eq.). The solution is adjusted with sodium hydroxide solution to a final pH of 9.0 – 9.2 which initiates precipitation of **S6** as a Mg^{2+}-complex. The mixture was incubated at 4 °C overnight. The precipitate is pelleted by centrifugation (3000 g, 15 min) in a 50 ml tube and washed twice with 20 ml of 8 mM $MgCl_2$ solution at pH 9.0. The resulting pellet is resuspended in 10 ml of water resulting in a milky solution without any clumps. Meanwhile 15 ml bed volume of Amberlite® IRC-748 (chelating) ion exchange resin (Alfa Aesar, L19570), which was washed in advance extensively with deionized water and methanol and stored in methanol until use) is loaded into a 20 ml peptide reactor column (or another small column) and equilibrated by passing through 100 ml of water. 8 ml bed volume of this Amberlite are added to the $InsP_6$ suspension and incubated at rt on a shaking platform for 30 min until the supernatant turns clear. The content of the tube was transferred onto the remaining Amberlite column and the eluate (gravity-flow) was collected. Additional 20 ml of water pushed through the Amberlite column and the eluates are combined and lyophilized. The resulting clean material was redissolved in D_2O for analysis, filtered through a 0.2 µm PTFE syringe filter and pH was adjusted by addition of DCl solution to 7.0 and dilution to a defined volume. The concentration of 1[$^{13}C_1$]$InsP_6$ was determined against a quantitative NMR-standard (phosphonoacetic acid). In total 0.105 mmol (91%) of clean 1[$^{13}C_6$]$InsP_6$ were obtained.

1[$^{13}C_6$]$InsP_6$ (S6):

^{1}H NMR (600 MHz, Deuterium Oxide) δ 4.90 (dq, *J* = 7.7, 2.5 Hz, 1H, 2-position), 4.39 (qt, *J* = 9.5, 2.9 Hz, 2H, 4/6-position), 4.11 (q, *J* = 9.6 Hz, 1H, 3-position), 4.09 (t, *J* = 9.5 Hz, 1H, 5-position), 4.09 (dt, *J* = 144.0, 9.4 Hz, 1H, 1-position).

^{13}C NMR (151 MHz, D_2O) δ[ppm]: 80.16, 78.69, 76.91, 76.24 (1-position).

^{31}P NMR (243 MHz, D_2O) δ 1.94, 1.08, 0.73.

HRMS *m/z*: $[M – 2H]^{2-}$ calcd. for $^{13}C_1{}^{12}C_5H_{16}O_{24}P_6$ 329.4251; found 329.4242.

Cloning, expression and purification of recombinant human MINPP1

A gene sequence encoding for human MINPP1 (29-487, Uniprot Q9UNW1-1) lacking the N-terminal signal peptide was designed and ordered using Thermo Fisher's GeneArt service. The sequence was codon-optimized for expression in *E. coli* and contains a NdeI (at initial ATG) and XhoI (after the stop codon) restriction site. The MINPP1 gene was cloned into the vector pET-15b using the NdeI and XhoI restriction sites. The resulting plasmid (pET-15b-MINPP1) encodes an N-terminal His-tag with a thrombin cleavage site followed by MINPP1. For plasmid preparation the *E. coli* Top10 strain was used.

The complete nucleotide sequence of the ORF of pET-15b-MINPP1 is as follows:

CC**ATGGGCAGCAGCCATCATCATCATCATCACAGCAGCGGCCTGGTGCCGCGCGGCAGCCATAT**
GCGTTGTAGCCTGCTGGAACCGCGTGATCCGGTTGCAAGCAGCCTGAGTCCGTATTTTGGTACAA
AAACCCGTTATGAAGATGTGAATCCGGTTCTGCTGAGCGGTCCGGAAGCACCGTGGCGTGATCCT
GAACTGCTGGAAGGCACCTGTACACCGGTTCAGCTGGTTGCACTGATTCGTCATGGCACCCGTTA
TCCGACCGTTAAACAAATTCGTAAACTGCGTCAGCTGCATGGTCTGCTGCAGGCACGTGGTAGCC
GTGATGGTGGTGCCAGCAGCACCGGTAGTCGTGATCTGGGTGCAGCACTGGCAGATTGGCCTCT
GTGGTATGCAGATTGGATGGATGGTCAGCTGGTAGAAAAAGGTCGTCAGGATATGCGTCAACTG
GCACTGCGTCTGGCAAGCCTGTTTCCGGCACTGTTTAGCCGTGAAAATTATGGTCGTCTGCGTCT
GATTACCAGCAGCAAACATCGTTGTATGGATAGCAGCGCAGCATTTCTGCAAGGTCTGTGGCAGC
ATTATCATCCGGGTCTGCCTCCGCCTGATGTTGCAGATATGGAATTTGGTCCGCCTACCGTTAATG
ATAAACTGATGCGTTTTTTTGACCATTGCGAGAAGTTTCTGACCGAGGTTGAAAAAAATGCAACCG
CACTGTATCATGTGGAAGCATTTAAAACAGGTCCGGAAATGCAGAACATCCTGAAAAAAGTTGCA
GCAACCCTGCAGGTTCCGGTTAATGATCTGAATGCCGATCTGATTCAGGTTGCCTTTTTTACCTGT
TCATTTGACCTGGCCATTAAAGGTGTTAAAAGCCCGTGGTGTGATGTGTTTGATATTGATGATGCA
AAGGTGCTGGAATATCTGAACGATCTGAAACAGTATTGGAAACGCGGTTATGGCTATACCATTAA
TAGCCGTAGCAGCTGTACCCTGTTTCAGGATATTTTTCAGCATCTGGATAAAGCCGTTGAACAGAA
ACAGCGTAGCCAGCCGATTAGCAGTCCGGTTATTCTGCAGTTTGGTCATGCGGAAACCCTGCTGC
CGCTGCTGAGCCTGATGGGTTATTTCAAAGATAAAGAACCGCTGACCGCCTACAACTATAAAAAG
CAGATGCATCGTAAATTTCGCAGCGGTCTGATTGTTCCGTATGCAAGCAATCTGATTTTTGTGCTG
TATCATTGCGAAAATGCGAAAACCCCGAAAGAACAGTTTCGTGTTCAGATGCTGCTGAATGAAAA
AGTTCTGCCGCTGGCATATAGCCAAGAAACCGTTAGCTTTTATGAGGACCTGAAAAACCACTACA
AAGATATCCTGCAGAGCTGTCAGACCAGCGAAGAATGTGAACTGGCACGTGCAAATAGCACCAG
TGATGAACTGTAACTCGAGGATCC

Complete ORF of pET-15b-MINPP1. Restriction sites are highlighted (NcoI in yellow, NdeI in green, XhoI in light blue). The sequence encoding MINPP1 is shown in bold and the font colour for chosen component of the protein are changed (His-tag in blue, thrombin cleavage site in orange, catalytic histidine in red).

For protein expression *E. coli* BL21 (DE3) was used which was transformed with the MINPP1-encoding plasmid using the heat-shock method. A 5 ml-overnight culture of the transformed bacterial strain in terrific broth (TB, Formedium) and Ampicillin (100 µg/mL, Roth) at 37 °C was inoculated into 500 ml of TB and Ampicillin. The culture was cooled to 18 °C when OD_{600nm} = 0.5 was reached (~160 min after inoculation). Protein expression was induced at OD_{600nm} = 0.6 (~170 min after inoculation) with 0.6 mM Isopropyl β-D-1-thiogalactopyranoside (IPTG, Thermo Scientific). The culture was incubated at 18 °C for 18-20 h. The bacterial suspension was centrifuged (3000 g, 15 min, 4 °C) upon which a bacterial pellet of ~1.5 g wet

weight was obtained. The pellet was resuspended in 50 ml ice-cold lysis buffer (150 mM NaCl, 10 mM Tris*HCl (Roth), pH 8.0, 1 mM DTT (Roth or VWR), 1X cOmplete™ protease inhibitor cocktail (Roche)) and a spatula tip of lysozyme (Roth) and DNAse I (Roche) were added. The bacterial cells were lysed using a homogenizer (LM10 Microfluidizer, Microfluidics, 15000 psi, 5 passages). The resulting suspension was centrifuged (20 000 g, 20 min, 4 °C). The supernatant was used for purification of soluble MINPP1 while the resulting pellet was used for MINPP1 isolation from inclusion bodies (see below). Inclusion body-purification of MINPP1 yielded higher amounts.

The purification of soluble MINPP1 was adapted from Craxton *et al.* [7]: The supernatant was filtered (VWR vacuum filter, PES 0.45 µm) and the flowthrough was applied to a 5 ml Ni-NTA column (GE, HiTrap IMAC FastFlow) on a FPLC system (NGC Quest 10 Chromatography System, Bio-Rad) equilibrated to buffer A (150 mM NaCl, 10 mM Tris*HCl, pH 8.0, 1 mM DTT). The column was subsequently washed with 5 column volumes (CV) buffer A, 5 CV buffer A:B 10:7, 5 CV buffer B (1 M NaCl, 10 mM Tris*HCl, pH 8.0, 1 mM DTT), 5 CV buffer A with 2% buffer C (buffer C is identical to buffer A containing additional 500 mM imidazole (AppliChem), pH 8.0). For elution a gradient of 2% buffer C in A to 75% buffer C in A over 20 CV was applied. Fractions containing MINPP1 were united, concentrated using centrifugal filters (15 ml 10 kDa MWCO, Amicon Ultra) and dialyzed against 1 L of dialysis buffer 1 (150 mM NaCl, 10 mM Tris*HCl, pH 8.0, 1 mM DTT, 10 Vol-% glycerol (Roth), 0.25% CHAPS (Roth)) twice for 1.5 h. Protein concentration was determined using a BCA assay kit (Pierce™ BCA Protein Assay Kit). Protein solution was aliquoted and stored at -80 °C. However, soluble MINPP1 was obtained in only low amounts this way (2 mg from 1 L culture) which is a known problem with heterologous expression of MINPP1.[8]

For purification of MINPP1 from inclusion bodies: After removing the lysate, the pellet was washed by thoroughly resuspending in 35 ml ice-cold deionized water, centrifugation (20 000 g, 20 min, 4 °C) and after discarding the supernatant the resulting pellet was washed in the same manner two more times after which a pellet of 1.4 g wet weight was obtained. Per 0.7 g pellet mass, the pellet was resuspended in 30 ml resolubilization buffer (0.2 w/v-% *N*-lauroylsarcosin sodium salt (Sarkosyl, Fisher Scientific), 10 mM Tris*HCl, pH 8.0, 1 mM DTT). The suspension was incubated overnight at 4 °C in a 50 ml-tube under light agitation on a reciprocal shaker. The tube was centrifuged (3000 g, 30 min, 4 °C). 20 ml of recovered supernatant containing MINPP1 was dialyzed first against 1 L dialysis buffer 2 (150 mM NaCl, 10 mM Tris*HCl, pH 8.0, 1 mM DTT, 10 Vol-% glycerol, 0.1 % Triton-X100 (Roth)) for 3 h at 4 °C and then again against fresh dialysis buffer overnight at 4 °C. Protein concentration was determined using a BCA assay kit (Pierce™ BCA Protein Assay Kit). The dialyzed protein solution was adjusted to a final glycerol content of 30 Vol-%, aliquoted, flash-frozen in liquid nitrogen and stored at -80 °C. Using this inclusion body purification procedure 73 mg MINPP1 could be obtained from half of a 500 mL culture.

The activity of MINPP1 preparations were validated against its substrates 2,3-bisphophoglycerate (BPG), $InsP_5$[2OH] and $InsP_6$ using a Malachite green assay. The activity of inclusion body-purified MINPP1 against BPG was determined to be 22 nmol min^{-1} mg^{-1} enzyme, which is comparable to the value 16 nmol min^{-1} mg^{-1} enzyme reported in the literature.[2] The activity of soluble MINPP1 and inclusion-body purified MINPP1 did not differ drastically (see also Figure S8).

No decrease in activity was observed after over a year of storage (without freeze-thaw cycles).

Alternative solubilization buffers

Different solubilization buffers were also tested for the inclusion body purification of MINPP1 on a smaller scale. Several mild solubilization buffers [9] were unable to sufficiently resolubilize MINPP1 (40 mM TrisHCl, pH 8, with either 5 Vol-% DMSO or 5 Vol-% *n*-propanol (VWR); 90 mM TrisHCl, pH 8.6, 2 M urea). Among the resolubilization buffers only the following managed to solubilize MINPP1: 40 mM TrisHCl, pH 8 with a) 0.2% sarkosyl, b) 8 M urea or c) 6 M guanidinium chloride (see Figure S7).

The resolubilized MINPP1 solution from a) and b) were dialyzed against dialysis buffer with and without Triton-X. In general, it was observed that protein concentration with urea was higher than with Sarkosyl

(~1 mg/mL vs. ~3 mg/mL) and with Triton-X-containing dialysis buffer the protein yield was also slightly higher by ~0.2mg/mL.

For all *in vitro* experiments MINPP1 preparations were used based on resolubilization with sarkosyl and Triton-X-containing dialysis buffer (see above).

NMR-based enzymatic assays

For the *in vitro* dephosphorylation of $InsP_6$ and $InsP_5$[2OH] by **MINPP1** the following conditions were used unless stated otherwise:

The reaction buffer contained 100 mM NaCl, 100 mM Na_2SO_4, 25 mM HEPES, pH* = 7.4, 1 mM DTT, 1 mM EDTA (Sigma), 0.2 mg/mL BSA (Roth), 2 mM CHAPS, 175 µM (or 50 µM) of inositol phosphate substrate, 0.5 µM enzyme. The reactions were carried out in D_2O. For each sample (500 µL final volume), the reaction mixture was prepared without InsP substrate in a 1.5 ml microcentrifuge tube, prewarmed to 37 °C for 5 min before the reaction was started by adding the substrate. The reactions were quenched by boiling at 95 °C for 5 min. NMR spectra were recorded without further workup. For the substrate inhibition experiments, $InsP_5$[2OH] was mixed with aliquots of a dilution series of $InsP_6$ prior to addition to the reaction mixture.

Malachite green-based enzymatic assays

For comparing the enzymatic activities of MINPP1 preparations the dephosphorylation of BPG, $InsP_5$[2OH] and $InsP_6$ were compared. The reaction buffer contained 100 mM NaCl, 100 mM Na_2SO_4, 25 mM HEPES, pH* = 7.4, 1 mM DTT, 1 mM EDTA, 0.2 mg/mL BSA, 2 mM CHAPS, 0.5 µM enzyme. 5 mM 2,3-BPG, or 50 µM InsP, respectively, were used and the reaction was carried out in Milli-Q® water (50 µL total volume per sample) at 37 °C in 0.2 mL tubes. The reaction mixtures lacking MINPP1 were preincubated at 37 °C for 5 min and reactions were started by addition of MINPP1. After 15-25 min (BPG and $InsP_5$[2OH]) and 19 h ($InsP_6$), 20 µL of the reaction mixture were transferred into a clear, flat-bottom 96-well plate, each well already containing 20 µL of Malachite green assay solution (Sigma) and 40 µL Milli-Q® water. After incubation for 30 min at room temperature, absorption was measured at 620 nm on a TECANInfinite 200 Pro M-Plex Plate Reader. For calculating the amount of released phosphate, a dilution series of inorganic phosphate standard was measured in parallel with the same buffer background and no-enzyme controls (MINPP1 preparations did not show any phosphate background). All samples were prepared in triplicate.

For determining IC_{50} values for the inhibition of MINPP1-mediated dephosphorylation of $InsP_5$[2OH] by $InsP_6$. Same buffer used as described above but 0.1 µM enzyme was used. $InsP_5$[2OH] concentrations were either 70 µM or 20 µM and $InsP_6$ concentrations ranged from 12.8 µM to 0.025 µM in a two-fold serial dilution and 0 µM $InsP_6$. Phosphate release was measured as stated above but 40 µL reaction mixture were used. The samples were quenched after 1 h (70 µM substrate) or 25 min (20 µM substrate). For calculating the amount of released phosphate, a dilution series of inorganic phosphate standard was measured in parallel with the same buffer background and no-enzyme control). All samples were prepared in triplicate.

Mammalian cell culture and metabolic labeling

HT29 WT cells were a kind gift from the lab of Jan Carette[10] (William Kaiser laboratory, RRID: CVCL_0320). HEK293 cell lines (WT and *MINPP1*$^{-/-}$) were a kind gift of the labs of Adolfo Saiardi and Vincent Cantagrel and the generation of the *MINPP1*$^{-/-}$ cells was described previously.[11] The absence of MINPP1 in *MINPP1*$^{-/-}$ cells was verified by Western blots (see Figure S17). HCT116 were obtained from ATCC. H1975 cells were a kind gift of the Klingmüller lab (originally ATCC, CRL-5908).

Unless stated otherwise all cell lines are cultivated in DMEM (Gibco DMEM high glucose, no glutamine, product no. 11960044) supplemented with streptomycin/penicillin (100 U/mL final concentration, Gibco), L-glutamine (1X, Gibco GlutaMAX™) and 10% FBS (Pan Biotech), at 37 °C in an atmosphere with 5% CO_2, and 95% humidity.

The metabolic labeling was conducted as described in a previous publication.[5] Briefly, cells are seeded at a density of $3\cdot10^5$ on a 15 cm culture dish in custom DMEM containing no regular inositol nor FBS but 100 µM [$^{13}C_6$]*myo*-inositol (or the respective isotopomer) and 10% dialyzed FBS (Gibco, product no. 26400044) instead (from here on referred to as “labeling medium”). Upon reaching ~85 % confluency, the cells are split into five 15 cm culture dishes in labeling medium. Upon reaching confluency cells were harvested by trypsination, collected in 50 ml tubes and washed twice with 50 ml ice-cold PBS or 0.9% NaCl solution. Packed cell volumes were determined for quantification. The collected cell pellets were either processed immediately after harvest or flash-frozen and stored at -80 °C. Metabolites were extracted by $HClO_4$-extraction. Lyophilized metabolite extracts were redissolved in D_2O, re-lyophilized, and finally measured in D_2O (dry D_2O from ampulla, Eurisotop D215T). For quantification 100 µM TMPBr was added to each sample. Standard curves for $InsP_6$ and $InsP_5$[2OH] concentrations against TMPBr are reported previously.[5] The concentrations of other ^{13}C-labeled InsP species for which no labeled standards were available were estimated using the standard curve for $InsP_6$. Cellular InsP concentrations were backcalculated from PCV.

TiO_2 enrichment of InsPs for NMR samples was adapted from published procedures.[12] Briefly, 500 µL of InsP containing sample is mixed 1:1 with ice-cold 1 M aq. perchloric acid and incubated for 30 min on ice (frozen samples are thawn directly in the perchloric acid and then incubated on ice). The sample is then centrifuged (10 min, 18 000 g, 4 °C) and the supernatant transferred into a separate 1.5 mL tube containing 5 mg of TiO_2 beads (Titanosphere 5 µm, GL Sciences), which were already washed with 500 µL Milli-Q® water and 500 µL 1 M perchloric acid ($HClO_4$, Supelco). The extract and TiO_2 beads were mixed on a rotary shaker on low speed for 5 min at 4 °C. The beads were briefly washed twice with 500 µL ice-cold 1 M perchloric acid (note: For centrifugation a table centrifuge (IKA miniG, 6000 rpm) was used at 1 min, and for transferring the supernatant without disturbing the TiO_2 beads a 2 µL Eppendorf tip attached to the tip of a 1 mL tip was used). Supernatans were united to check for unbound InsP species, neutralized roughly with 750 µL 2 M potassium hydroxide, centrifuged and the supernatant lyophilized. To eluate InsPs from the TiO_2 beads, the beads were incubated with 250 µL of 10% ammonia solution for 5 min at rt on a rotary shaker. After centrifugation the supernatant was collected in a separate tube. The elution step is repeated once more and the eluates are combined. The combined eluates are filtered through a 0.2 µM syringe filter (Sartorius Minisart RC4) which was subsequently rinsed with 150 µL of Milli-Q® water. The filtrate was collected in a new 1.5 mL tube and lyophilized. To reduce the water content for NMR analysis the lyophilized eluates were redissolved in 500 µL D_2O and lyophilized again. For NMR measurement the eluates are redissolved in 500 µL D_2O, pH* was adjusted to 6.0.

For the **metabolic flux analysis** *via* CE-MS HEK293 cells were first metabolically labeled with [$^{13}C_6$]Ins as described above over two passages. One week prior to harvest, $4\cdot10^5$ of the [$^{13}C_6$]Ins-labeled cells were seeded into one 15 cm dish per time point in [$^{13}C_6$]Ins-labeling medium. For each time point (72, 48, 24, 18, 12.5, 8, 4, 2 and 1 h before harvest) one plate had its medium removed and washed once with 0.9% NaCl solution. The cells were then continued to incubate in 4,5[$^{13}C_2$]Ins-containing labeling medium. For harvesting, the cells of one plate were washed with 25 mL 0.9% NaCl solution, trypsinized (3 mL), then resuspended in 7 mL 0.9% NaCl solution, then pelleted, washed once with 15 mL 0.9% NaCl per pellet and kept on ice until flash-freezing and storage at -80 °C until further processing. For preparing CE-MS each cell pellet was processed as follows: Cells were lysed by resuspending in 1 mL ice-cold 1 M $HClO_4$ (4 °C, 10 min) and centrifuged (18 000 g, 5 min, 4 °C). The supernatant was added to 4 mg of TiO_2 beads (prepared as described above) and the TiO_2 enrichment protocol was followed as described above until the first lyophilization step. Lyophilized samples were stored at -20 °C until CE-MS measurement. CE-MS measurements were carried out as described above. InsP-isotopomers were quantified relatively to each other.

Subcellular organelle isolation

For isolating cellular organelles, wild-type HEK293T were grown in complete DMEM up to 80% confluency. The cells were then harvested by scraping (cell scraper VWR, 734-2604) and were washed thrice with PBS by centrifugation at 250 g for 5 mins at 4 °C. The cell pellet was then processed for organelle isolation

using the respective protocols as mentioned below. All the buffers in the following protocols contain protease inhibitors (5 mM Benzamidine and 20 µg/mL pepstatin). Once isolated, the protein concentration was measured using a BCA assay kit following brief sonication. The organelle preparations were then stored at -80 °C until further use. Results are shown in Figure S17.

ER (microsome) Isolation

Intact microsomes were isolated according to published procedures with slight modifications.[13] Briefly, harvested cells we suspended in 2 mL SH buffer (0.25 M sucrose, 5 mM HEPES, pH 7.4) and homogenized in a dounce homogenizer with a clearance of 0.15 – 0.2 mm (tight fitting) with ten strokes. The lysate was centrifuged at 6000 g for 5 mins and the pellet was discarded. The supernatant was centrifuged at 15 000 g for 5 mins. The supernatant was transferred to a new tube while the pellet was resuspended in fresh buffer and centrifuged again at 15 000 g for 5 mins. The two supernatants were pooled and centrifuged at 105 000 g for 40 mins. The pellet was resuspended in 100 µL of SH buffer and stored at -80 °C for further use.

Mitochondria Isolation

Mitochondria were isolated following published procedures with some modifications.[14] Briefly, the harvested cell pellet was weighed and 1 mL cold T-K-Mg buffer (10 mM Tris-HCl pH 7.4, 10 mM KCl, 0.5 mM $MgCl_2$) per 0.15 g of cell pellet was used for resuspending the cells. The suspension was incubated on ice for 10 mins and passed through a 5 µm syringe filter to lyse the cells. Sucrose stock solution (1 M sucrose, 10 mM Tris-HCl pH 7.4) was added immediately to achieve a final concentration of 0.25 M sucrose (3:1 lysed suspension : 1 M sucrose). The lysate was then centrifuged at 1200 g for 3 mins to pellet unbroken cells, nuclei, and other cellular debris. The pellet was discarded and this step was repeated until no pellet was visible. The supernatant was then centrifuged at 15 000 g for 5 mins to pellet the mitochondria. The supernatant was discarded and the pellet was resuspended in STE buffer (0.32 M sucrose, 1 mM EDTA, 10 mM TrisHCl pH 7.4). The pellet was washed with STE buffer twice at 15 000 g for increased mitochondrial purity. The pellet containing mitochondria was resuspended in minimal volume of STE buffer and stored at -80 °C for further use.

Nuclei Isolation

The protocol for isolating nuclei was adapted from Hymer *et al.*[15] with slight modifications. Briefly, cells were grown and harvested by scraping and washing with PBS with centrifugation at 250 g for 5 mins. The cell pellet was resuspended in ice-cold nuclear extraction buffer (320 mM Sucrose, 5 mM $MgCl_2$, 10 mM HEPES,pH 7.4 1% Triton X-100) and incubated on ice for 10 min with mild intermittent mixing. The suspension was centrifuged at 2000 g for 5 mins at 4 °C and the supernatant was discarded. The pellet was washed with nuclear wash buffer (320 mM sucrose, 5 mM $MgCl_2$, 10 mM HEPES, pH 7.4) by centrifugation at 2000 g for 5 mins at 4 °C. The nuclei were then stored at -80 °C for further use.

Western blots

For Western blots the following primary antibodies were used: MINPP1: MIPP(A-8) sc-514214 (Santa Cruz), Calnexin: Calnexin-HRP conjugate C5C9 #40090 (Cell signalling), Fibrillarin: Fibrillarin(B-1)-HRP conjugate sc-166001 (Santa Cruz), Citrate synthase: D7V8B #14309 (Cell signaling). Following secondary antibodies were used: Anti-rabbit IgG, HRP-linked 7074S (Cell Signaling), Anti-mouse IgG, HRP-linked 7076S (Cell Signaling).

For Western blot analysis 20 µg of the respective samples (except recombinant MINPP1, 0.02 µg) were subjected to SDS-PAGE (4-20% Mini-PROTEAN® TGX Precast gels, 10 well, BioRad, 60 - 90 min, 100V, in SDS running buffer (25 mM Tris, 192 mM glycine, 3 mM sodium dodecylsulfate). A Trans-Blot® SD system (BioRad) was used for transfer onto a nitrocellulose membrane. Standard immunoblotting techniques were applied afterwards.

References

(1) Puschmann, R.; Harmel, R. K.; Fiedler, D. Analysis of Metabolically Labeled Inositol Phosphate Messengers by NMR. In *Methods in Enzymology Vol ume 641*; 2020; pp 35–52. https://doi.org/10.1016/bs.mie.2020.04.035.

(2) Cho, J.; King, J. S.; Qian, X.; Harwood, A. J.; Shears, S. B. Dephosphorylation of 2,3-Bisphosphoglycerate by MIPP Expands the Regulatory Capacity of the Rapoport-Luebering Glycolytic Shunt. *Proc. Natl. Acad. Sci. U. S. A.* **2008**, *105* (16), 5998–6003. https://doi.org/10.1073/pnas.0710980105.

(3) Cheng, Y.-C.; Prusoff, W. H. Relationship between the Inhibition Constant (KI) and the Concentration of Inhibitor Which Causes 50 per Cent Inhibition (I50) of an Enzymatic Reaction. *Biochem. Pharmacol.* **1973**, *22* (23), 3099–3108. https://doi.org/10.1016/0006-2952(73)90196-2.

(4) Puschmann, R.; Harmel, R. K.; Fiedler, D. Scalable Chemoenzymatic Synthesis of Inositol Pyrophosphates. *Biochemistry* **2019**, *58* (38), 3927–3932. https://doi.org/10.1021/acs.biochem.9b00587.

(5) Harmel, R. K.; Puschmann, R.; Nguyen Trung, M.; Saiardi, A.; Schmieder, P.; Fiedler, D. Harnessing 13 C-Labeled Myo -Inositol to Interrogate Inositol Phosphate Messengers by NMR. *Chem. Sci.* **2019**, *10*, 5267–5274. https://doi.org/10.1039/C9SC00151D.

(6) Qiu, D.; Eisenbeis, V. B.; Saiardi, A.; Jessen, H. J. Absolute Quantitation of Inositol Pyrophosphates by Capillary Electrophoresis Electrospray Ionization Mass Spectrometry. *J. Vis. Exp.* **2021**, *2021* (174), 1–13. https://doi.org/10.3791/62847.

(7) Craxton, A.; Caffrey, J. J.; Burkhart, W.; Safrany, T. S.; Shears, B. S. Molecular Cloning and Expression of a Rat Hepatic Multiple Inositol Polyphosphate Phosphatase. *Biochem. J.* **1997**, *328* (1), 75–81. https://doi.org/10.1042/bj3280075.

(8) Cho, J.; Choi, K.; Darden, T.; Reynolds, P. R.; Petitte, J. N.; Shears, S. B. Avian Multiple Inositol Polyphosphate Phosphatase Is an Active Phytase That Can Be Engineered to Help Ameliorate the Planet's "Phosphate Crisis." *J. Biotechnol.* **2006**, *126* (2), 248–259. https://doi.org/10.1016/j.jbiotec.2006.04.028.

(9) Singh, A.; Upadhyay, V.; Upadhyay, A. K.; Singh, S. M.; Panda, A. K. Protein Recovery from Inclusion Bodies of Escherichia Coli Using Mild Solubilization Process. *Microb. Cell Fact.* **2015**, *14* (1), 1–10. https://doi.org/10.1186/s12934-015-0222-8.

(10) Dovey, C. M.; Diep, J.; Clarke, B. P.; Hale, A. T.; McNamara, D. E.; Guo, H.; Brown, N. W.; Cao, J. Y.; Grace, C. R.; Gough, P. J.; Bertin, J.; Dixon, S. J.; Fiedler, D.; Mocarski, E. S.; Kaiser, W. J.; Moldoveanu, T.; York, J. D.; Carette, J. E. MLKL Requires the Inositol Phosphate Code to Execute Necroptosis. *Mol. Cell* **2018**, *70* (5), 936-948.e7. https://doi.org/10.1016/j.molcel.2018.05.010.

(11) Ucuncu, E.; Rajamani, K.; Wilson, M. S. C.; Medina-Cano, D.; Altin, N.; David, P.; Barcia, G.; Lefort, N.; Banal, C.; Vasilache-Dangles, M. T.; Pitelet, G.; Lorino, E.; Rabasse, N.; Bieth, E.; Zaki, M. S.; Topcu, M.; Sonmez, F. M.; Musaev, D.; Stanley, V.; Bole-Feysot, C.; Nitschké, P.; Munnich, A.; Bahi-Buisson, N.; Fossoud, C.; Giuliano, F.; Colleaux, L.; Burglen, L.; Gleeson, J. G.; Boddaert, N.; Saiardi, A.; Cantagrel, V. MINPP1 Prevents Intracellular Accumulation of the Chelator Inositol Hexakisphosphate and Is Mutated in Pontocerebellar Hypoplasia. *Nat. Commun.* **2020**, *11* (1). https://doi.org/10.1038/s41467-020-19919-y.

(12) Wilson, M. S. C.; Bulley, S. J.; Pisani, F.; Irvine, R. F.; Saiardi, A. A Novel Method for the Purification of Inositol Phosphates from Biological Samples Reveals That No Phytate Is Present in Human Plasma or Urine. *Open Biol.* **2015**, *5* (3), 150014. https://doi.org/10.1098/rsob.150014.

(13) Sukhodub, A. L.; Burchell, A. Preparation of Intact Microsomes from Cultured Mammalian H4IIE Cells. *J. Pharmacol. Toxicol. Methods* **2005**, *52* (3), 330–334. https://doi.org/10.1016/j.vascn.2005.04.016.

(14) Choi, A.; Barrientos, A. Sucrose Gradient Sedimentation Analysis of Mitochondrial Ribosomes. In *Methods in Molecular Biology*; 2021; pp 211–226. https://doi.org/10.1007/978-1-0716-0834-0_16.

(15) HYMER, W. C.; KUFF, E. L. ISOLATION OF NUCLEI FROM MAMMALIAN TISSUES THROUGH THE USE OF TRITON X-100. *J. Histochem. Cytochem.* **1964**, *12* (5), 359–363. https://doi.org/10.1177/12.5.359.

NMR spectra

1[$^{13}C_1$]Ins (**S3a**):

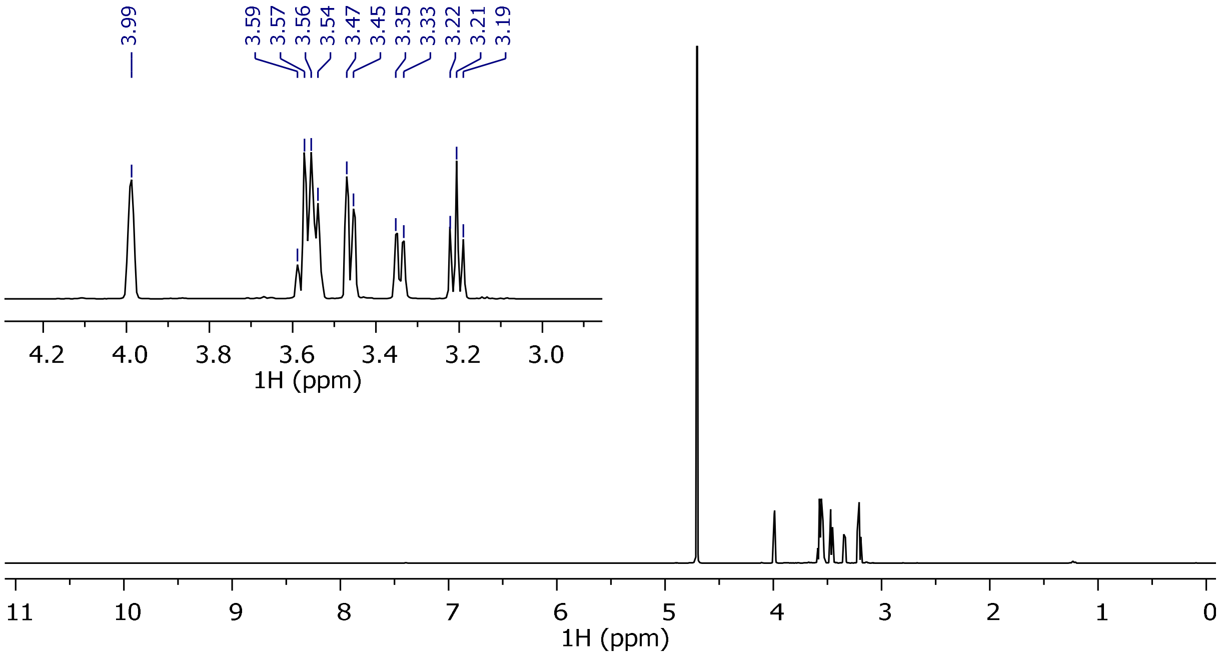

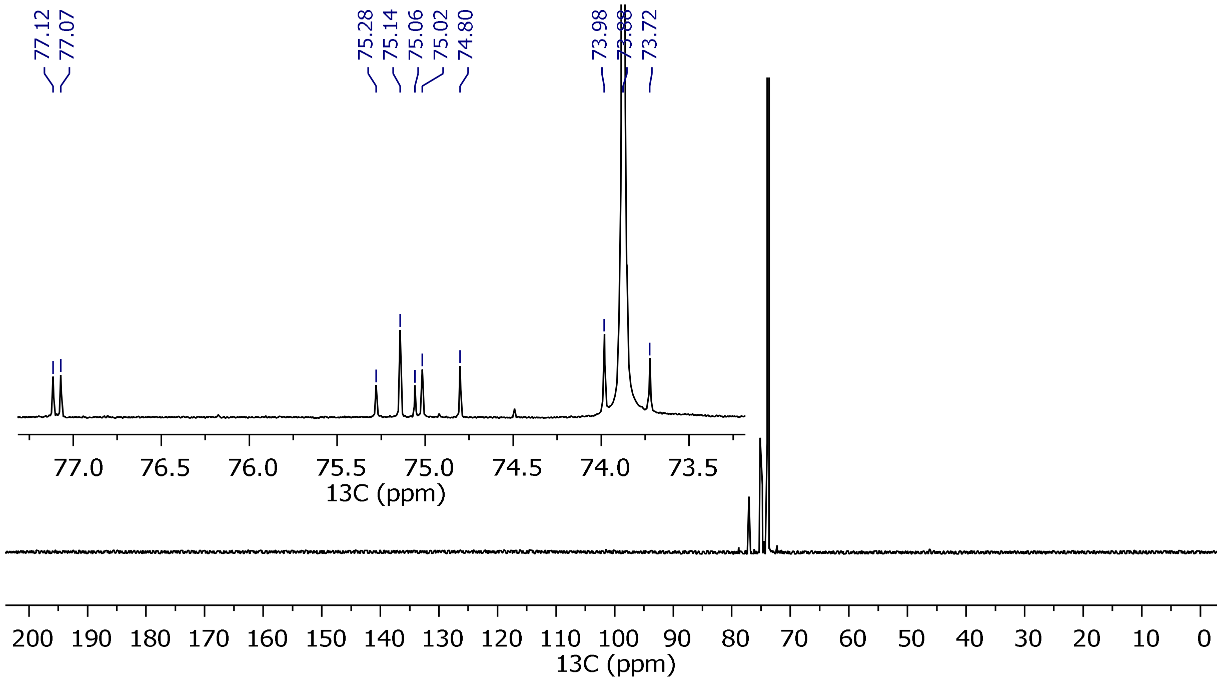

3[$^{13}C_1$]Ins (**S3a'**):

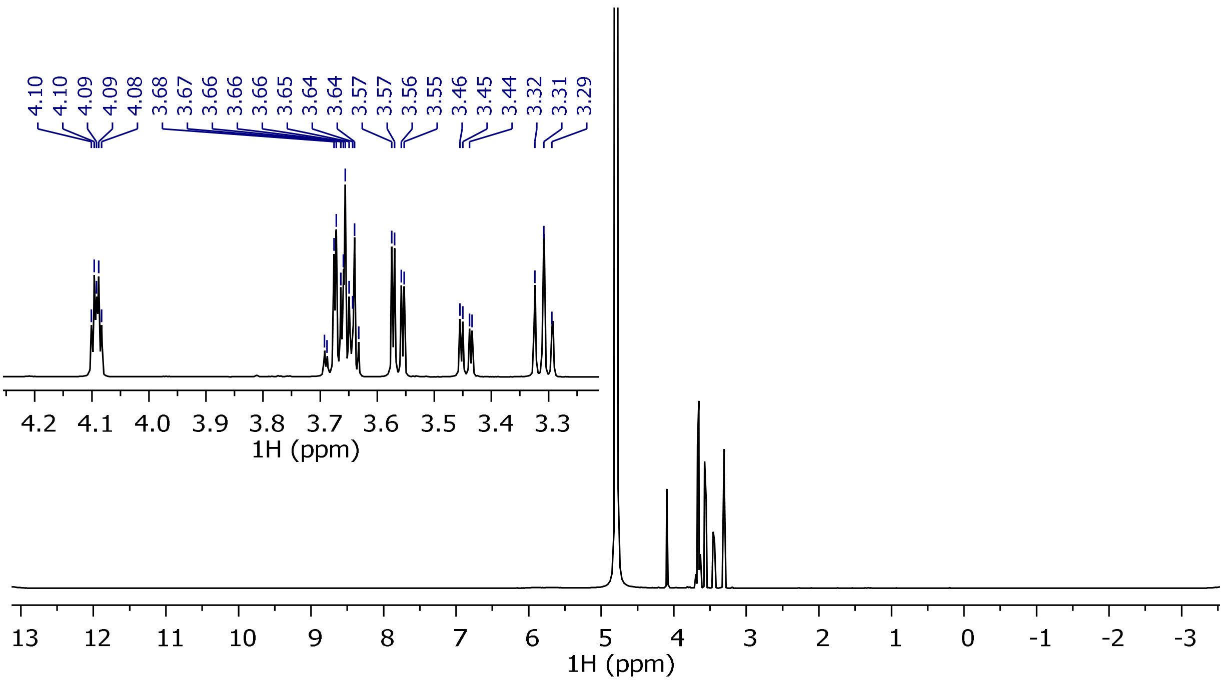

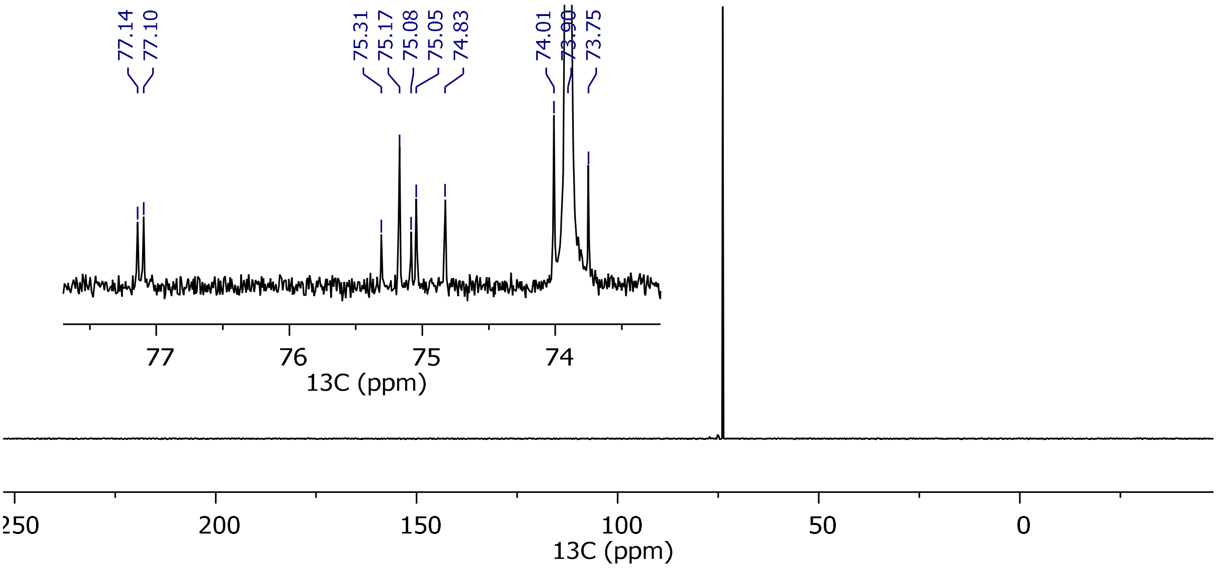

4,5[$^{13}C_2$]Ins (**S3b**):

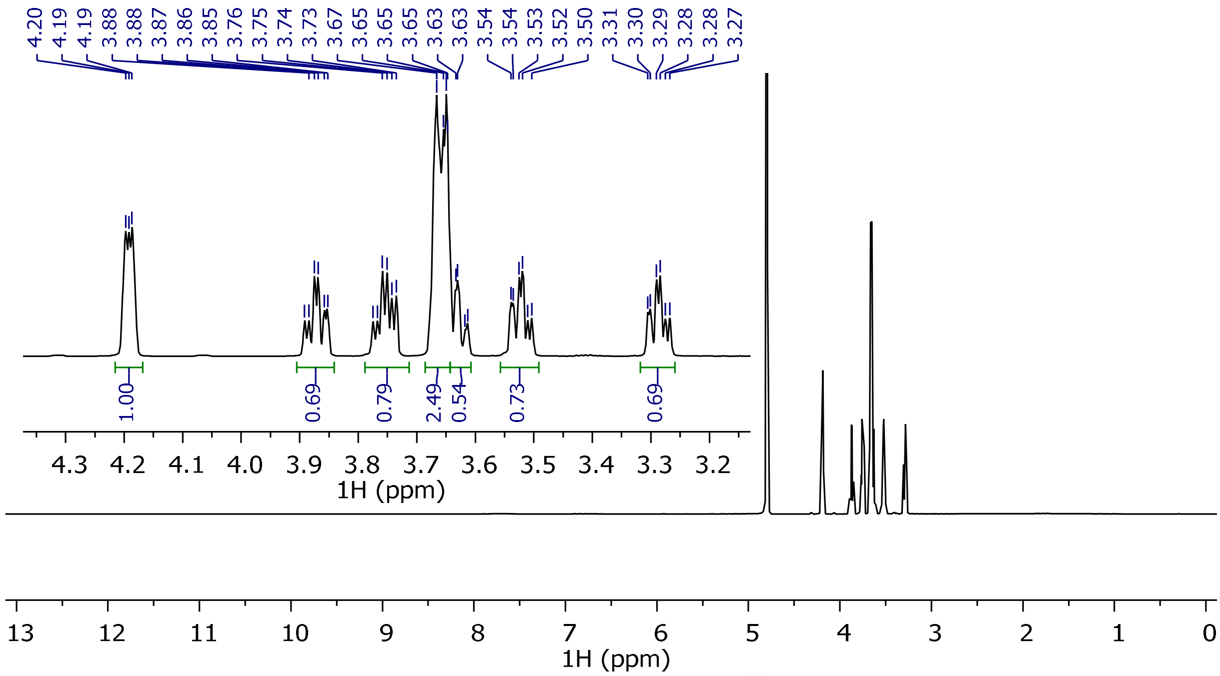

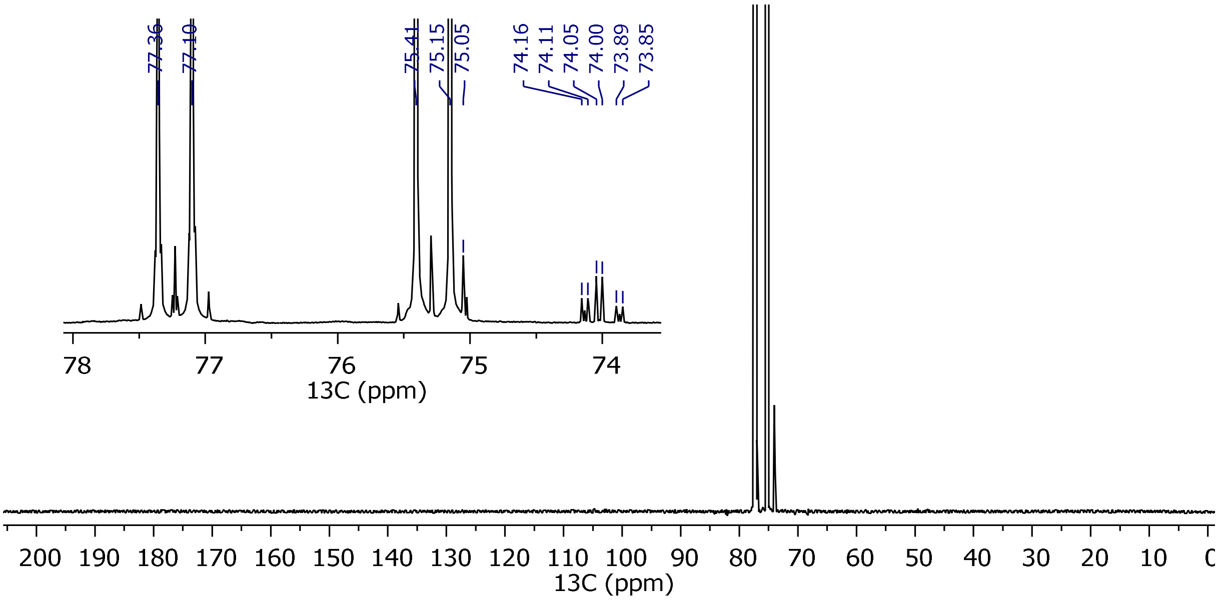

S5:

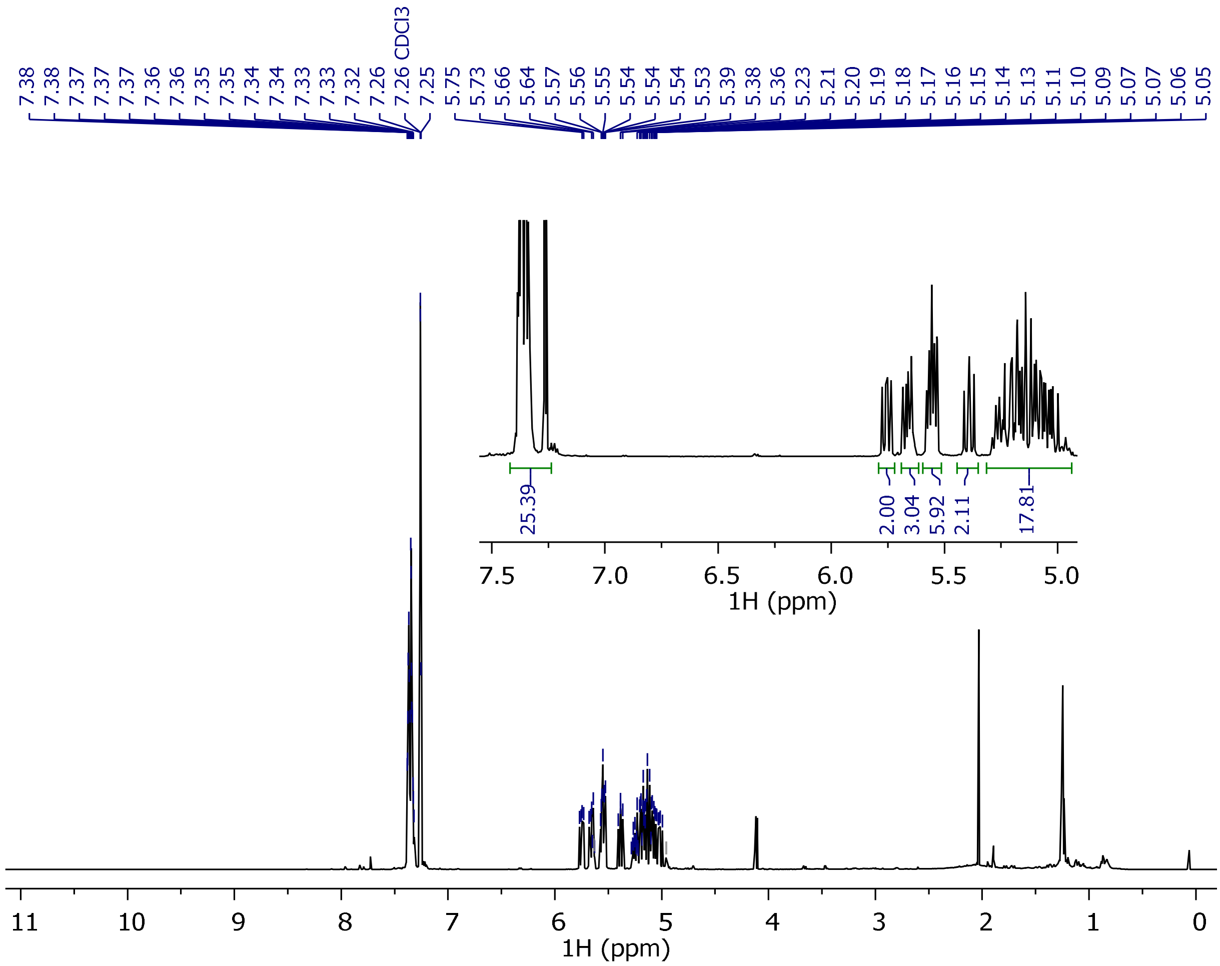

7.38
7.38
7.37
7.37
7.37
7.36
7.36
7.35
7.35
7.34
7.34
7.33
7.33
7.32
7.26
7.26 CDCl3
7.25
5.75
5.73
5.66
5.64
5.57
5.56
5.55
5.54
5.54
5.54
5.53
5.39
5.38
5.36
5.23
5.21
5.20
5.19
5.18
5.17
5.16
5.15
5.14
5.13
5.11
5.10
5.09
5.07
5.07
5.06
5.05
25.39
2.00
3.04
5.92
2.11
17.81
7.5
7.0
6.5
6.0
5.5
5.0
1H (ppm)
11
10
9
8
7
6
5
4
3
2
1
0
1H (ppm)

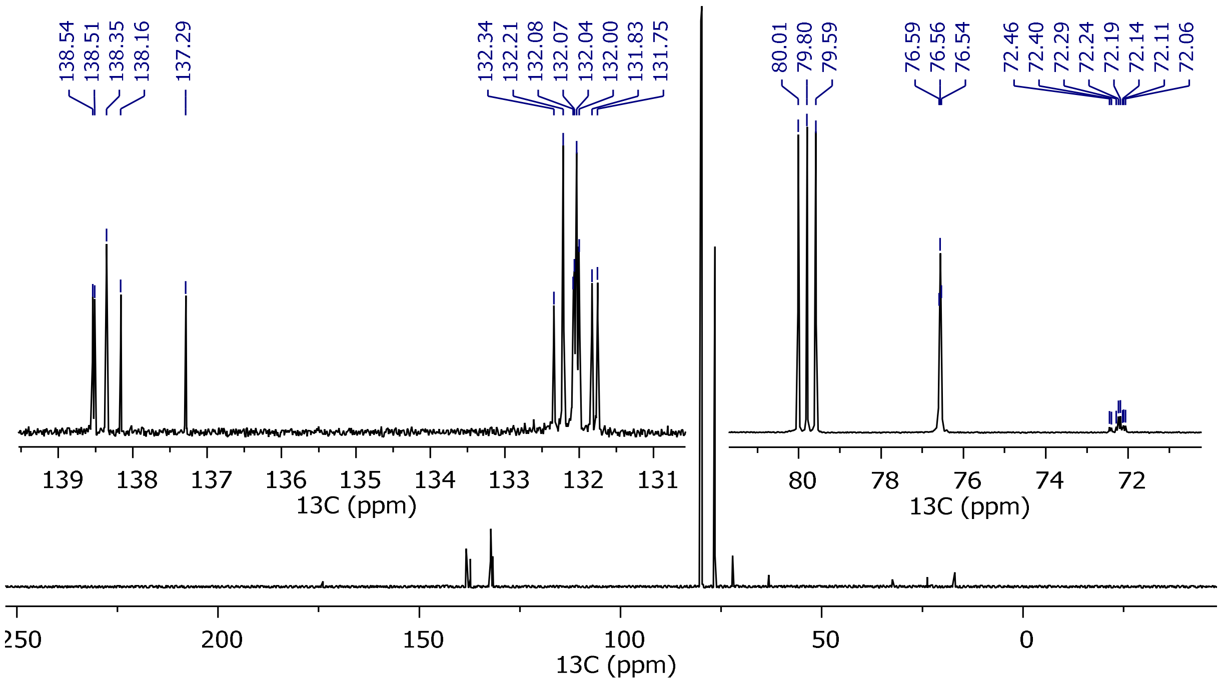
138.54
138.51
138.35
138.16
137.29
132.34
132.21
132.08
132.07
132.04
132.00
131.83
131.75
80.01
79.80
79.59
76.59
76.56
76.54
72.46
72.40
72.29
72.24
72.19
72.14
72.11
72.06
13C (ppm)
13C (ppm)
13C (ppm)

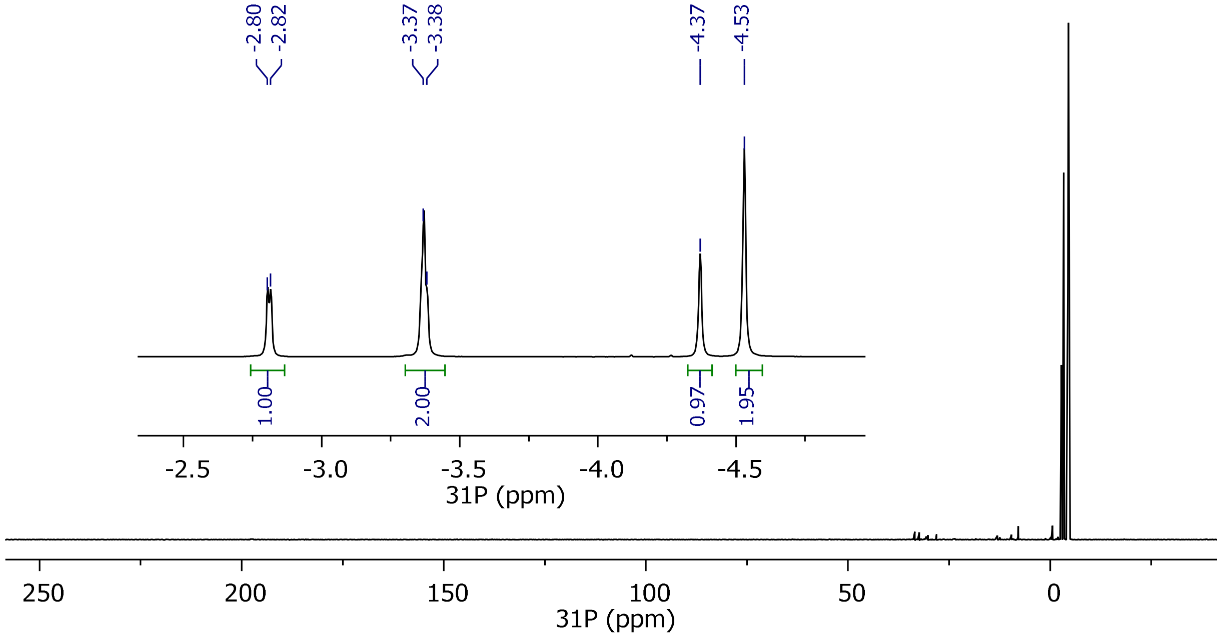
-2.80
-2.82
-3.37
-3.38
-4.37
-4.53
1.00
2.00
0.97
1.95
31P (ppm)
31P (ppm)

1[$^{13}C_6$]InsP$_6$ (**S6**):

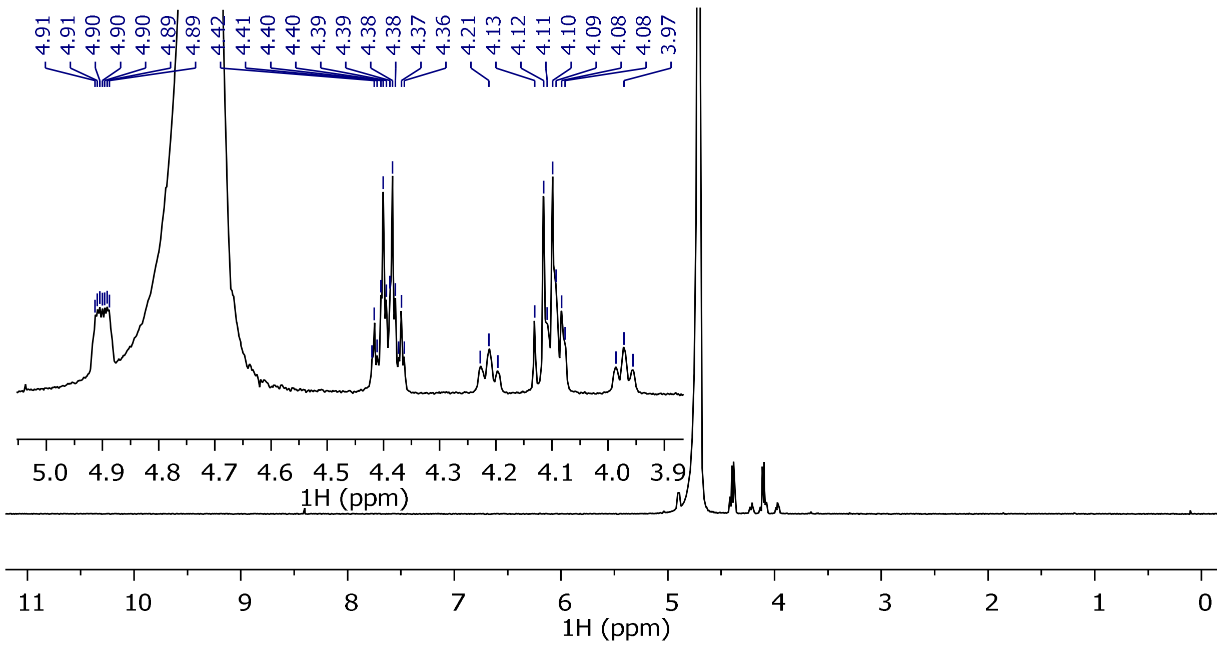

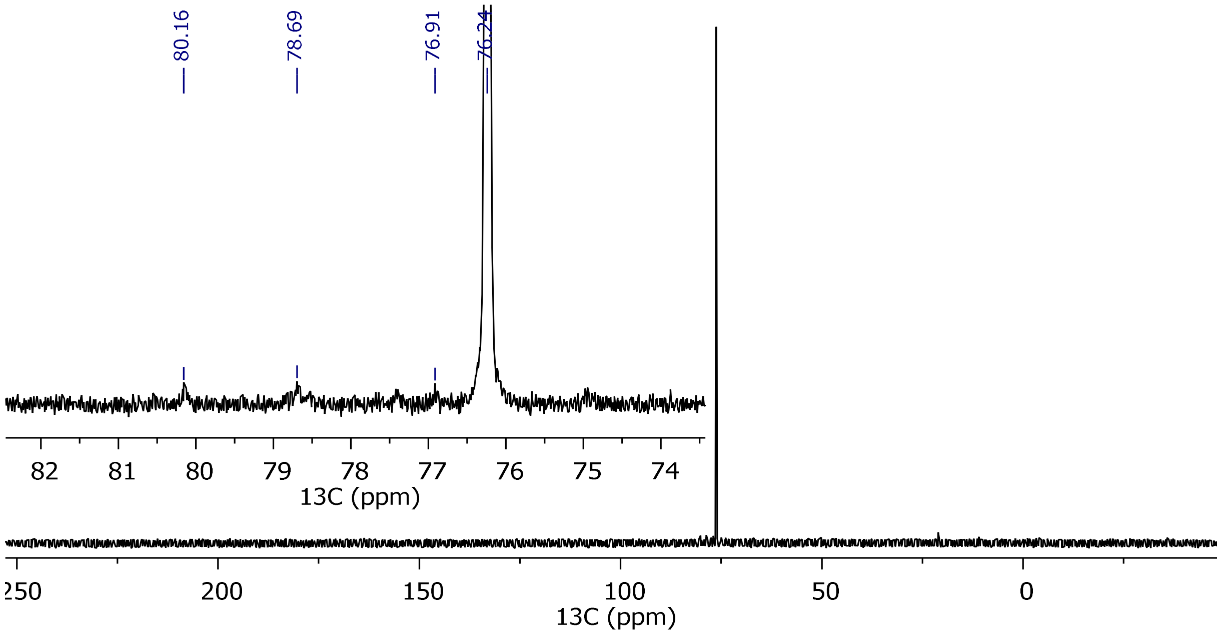

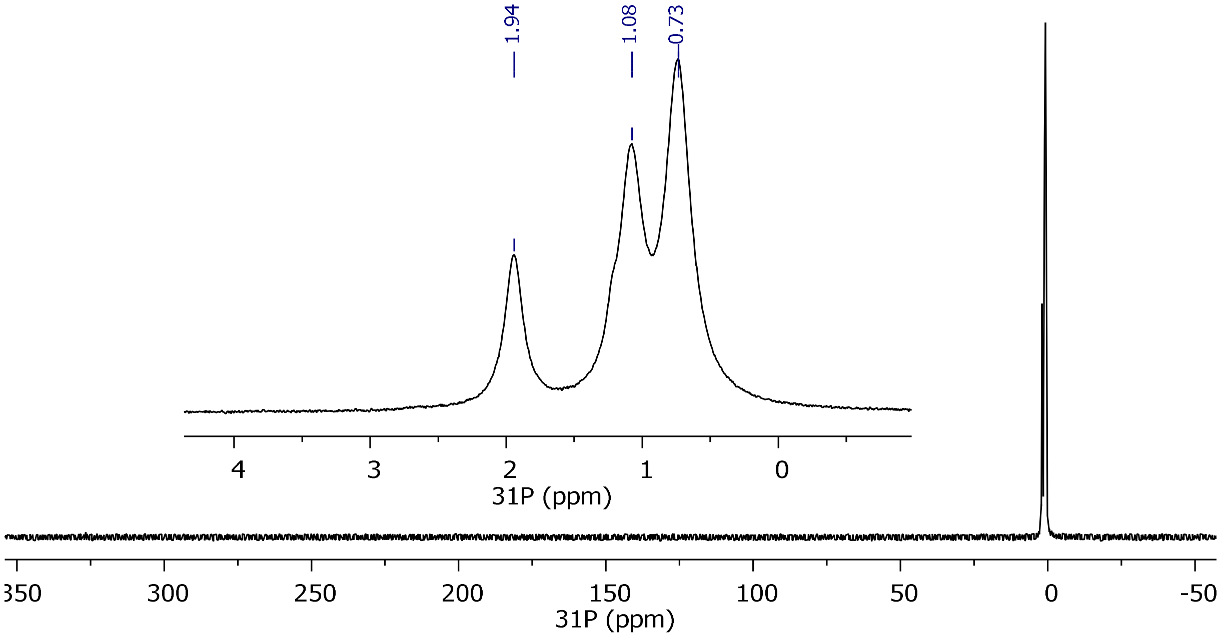
1.94
1.08
0.73
4
3
2
1
0
31P (ppm)
350
300
250
200
150
100
50
0
-50
31P (ppm)
