# Supplementary material for: Stable Isotopomers of myo-Inositol Uncover a Complex MINPP1-Dependent Inositol Phosphate Network
Source: ACS Cent Sci. 2022 Dec 5;8(12):1683–94. doi: 10.1021/acscentsci.2c01032 (PMC9801504; doi:10.1021/acscentsci.2c01032)
Supplement: Supplementary file 4 — oc2c01032_si_004.pdf [file oc2c01032_si_004.pdf]

oc-2022-01032p.R1

Name: Peer Review Information for "Stable isotopomers of *myo*-inositol uncover a complex MINPP1-dependent inositol phosphate network"

First Round of Reviewer Comments

Reviewer: 1

Comments to the Author

In this manuscript, Trung et al. describe a powerful 2D BIRD-{1H-13C}HMQC NMR method to detect and quantify metabolic labelling of 13C-tagged inositol species from cellular extracts. The authors demonstrate that by comparing to 13C-1H signals of inositol phosphate (InsP) standards, the same chemical species can be quantified following incorporation into multiple cell lines without need for chromatography- or electrophoresis-based separation. The authors apply this technique to address the InsP6-dephosphorylation pathway that produces lesser-phosphorylated metabolites following phosphorylation by a distinct synthesis pathway, a process that is as yet poorly understood. They identify from cellular extracts four major InsPs including InsP6 and Ins(1,3,4,5,6)P5, both of which were previously established to be abundant InsP metabolites, as well as distinguish between enantiomers of several such metabolites. The development and validation of this method is, from a technical point of view, outstanding. As a method, the isotopically labelled substrates and NMR analysis is an impressive achievement that will be useful for the field and a great illustration of how synthetic and analytical chemistry can be combined to inform on cellular metabolic pathways.

To demonstrate the utility of this method in elucidating metabolic pathways, the authors focus on the multiple inositol polyphosphate phosphatase 1 (MINPP1) as a target enzyme to further characterize its role in dephosphorylating both InsP6 and Ins(1,3,4,5,6)P5. Using kinetic assays of purified MINPP1 and 13C-labelled substrates, the authors characterize the activity of the enzyme to generate new chemical species that can be tracked via NMR. They establish kinetic parameters for the various dephosphorylation steps they observe, including the generation of two mostly overlooked members of the InsP family whose appearance in cells is eliminated in MINPP1-/- cells. However, the biological significance of these data remains unclear, including the physiological relevance of the kinetics data as it would apply in vivo, the relevance of the subsequent metabolic flux analysis in wild type and MINPP1-/- cells, and the physiological importance of the two InsP species that the authors focus on. Some or all of these issues merit substantial expansion. Additional specific comments follow:

Major comments:

1. A major concern with the results presented in figures 5, 6, 7, S7, S8, and S9 is that the kinetic parameters laid out in figure 6B would suggest that any catalytic activity of MINPP1 beyond the initial reactions with InsP5[2OH], InsP6, and possibly Ins(1,4,5,6)P4 are unlikely to be of biological significance. The rate constants for subsequent steps beyond those already characterized in the literature for

MINPP1 drop off by multiple log folds, resulting in the reaction rates measured in vitro over several days presented in these figures. Given the tremendous spread of rate constants, it is incumbent on the authors to provide substantial additional data to support the model that MINPP1 is the master regulator of all of these metabolic steps. Put another way, it is challenging to conceive that multiple reaction sequences involving more than 30 chemically distinct conversions would be catalyzed entirely by one enzyme in a cellular context, especially when it takes multiple days for these sequences to achieve more than 50% conversion in vitro. Considering also that lack of MINPP1 is not embryonically lethal but, much the opposite, its absence in MINPP1-deficient mice leads to minor or no phenotypic deficits (as reported in ref. 16), the role the authors suggest for MINPP1 in figure 7D and in the text as a key metabolic enzyme carrying out several steps of InsP6 dephosphorylation does not seem to be supported by the evidence presented. An equally compelling alternative hypothesis is that other enzymes, including the unidentified InsP6 phosphatase redundant with MINPP1's activity on this substrate (pg.12, line 8), might be involved in subsequent dephosphorylations to generate the lower phosphorylated inositol species the authors observed. Thus, as presented, the study does not convincingly show concordance between the in vitro kinetics and cellular knockout data, which are the two main pieces of data put forward to support the key model in figure 7.

2. Literature reports discussing MINPP1 (e.g. ref. 16) describe the role of the ER localization of the enzyme in its substrate specificity and role in inositol phosphate metabolism. The current manuscript does not directly address (except in the discussion) the compartmentalization of MINPP1 and the potential impact it would have on availability of the various InsP species the authors describe as being substrates in the dephosphorylation scheme. Questions of how the InsP substrates reach MINPP1 and in what proportions would merit much further discussion and representation in the schematics of Figure 1A and 7D.

Minor comments:

1. Figure 3C only includes one replicate for HT29 cells. Three replicates should be included to be able to compare these results to those from the other cell lines.

2. Figure 7B and 7C don't show all four metabolites in either model. The Ins(2,3)P2 in the MINPP1-/- cells would be especially useful for validating the data shown in figure 4B.

Comments by Line:

1. Pg. 4 line 54, "labelled" misspelled

2. Pg. 6 line 44, "extracts" misspelled

3. Pg. 9 line 32, extra space between 3 and %

Reviewer: 2

Comments to the Author

The manuscript by Trung, et al. applies 13C-NMR probes to quantify the abundance of various inositol phosphates in multiple human cells lines. Inositol phosphates are ancient and extremely important signaling molecules present in all forms of life, from viruses to bacteria to plants and humans, and thus

discoveries about such broadly important small biomolecules are of high impact and very broad interest. The authors here have made the unexpected discovery that several understudied forms of inositol phosphates are highly abundant in human cells, applying relatively new 13C-NMR metabolic labels to this long-standing problem. They go on to identify the enzyme responsible for the presence of these inositol phosphate species as MINPP1, using mammalian genetics, and elucidate the entire pathway for their synthesis by effectively applying computational metabolic flux analyses. The data strongly support all conclusions in the paper, and the conclusions do not overstate the data. Thus, this manuscript deserves publication after a few relatively minor problems have been addressed:

Major:

1) Kinetic analyses of MINPP1 use re-folded enzyme, given the unexpected nature of the IP6 curves some orthogonal confirmation of only the key aspects of these experiments using MINPP1 that has not been re-folded should be done. PTMs, etc, of eukaryotic sourced protein (if required) has its own caveats, but can also be denatured and refolded as a control.

2) Fig 6 shows IP6 inhibits MINPP1, and the mechanism is quite rightly suggested to be substrate competitive, but this is not demonstrated. IP6 should be more thoroughly characterized as a substrate competitive, non-competitive or uncompetitive inhibitor of MINPP1 activity on InsP5[2OH] dephosphorylation. If IP6 acts as a non-competitive or uncompetitive inhibitor, the impact of the work gets much higher.

3) Strong, orthogonal data support that Ins(2,3)P2 is high abundance in the two cell lines examined, impact would be enhanced if other cells were tested (e.g. yeast, bacteria, sf9 lepidopteran or even primary human hepatocytes) to broaden this conclusion. This would also better demonstrate flexibility of the 13C-NMR tools.

Minor:

4) In addition to reporting inositol phosphate concentrations using estimations of packed cell volumes, the authors should also report absolute quantifications to the internal standards somewhere in the paper.

5) Figure 3 concentration values also must estimate number of cells/genomes in the sample in addition to cell volumes to estimate the cellular concentration. These numbers should be included in main text of Fig 3 and in the legend.

6) No details on the MINPP1-KO cell line could be found in the supplemental. The genetics here are important because HEK293 cells often have more than two copies of entire chromosomes. How was MINPP1-KO established (western, pcr, etc)? How many copies of MINPP1 are present in WT HEK293 cells? Did the CRISPR hit all the alleles? Importantly, this is only a minor concern because regardless of the ploidy the conclusions still hold - even if the cells are MINPP1 -/+/+, the phenotype suggests even a small reduction in MINPP1 alters the relevant InsPs. This just needs clarification.

Author's Response to Peer Review Comments:

Please find attached our response to the decision letter.

Prof. Dorothea Fiedler, Ph.D.
Leibniz-Institut für Molekulare Pharmakologie
Robert-Rössle Str. 10
13151 Berlin, Deutschland
Tel.: +49 30 94793151
fiedler@fmp-berlin.de

November 7th, 2022

Dear Prof. Editor,

Thank you for considering our manuscript “Stable isotopomers of *myo*-inositol uncover a complex MINPP1-dependent inositol phosphate network” for publication in *ACS Central Science*. We were pleased to see the overall positive response from the two reviewers, and would like to thank them for their careful reading of the manuscript. Their comments and suggestions have been very helpful in assembling an improved, revised version. We also appreciated the comments from the editorial office and have checked all of those items and corrected them where necessary.

The revised manuscript is enclosed and has been modified to address the reviewers’ concerns; a detailed response to the individual points made by the reviewers can be found below. We have highlighted all changes in the revised manuscript. We are also including several revised Figures, additional Supporting Figures, an additional Supporting Table, as well as a revised Supporting Information. The revisions were supported by a co-worker from my group, and we have included him as an author.

Please do not hesitate to contact us if you or the reviewers have any additional questions.

D. Fiedler

Dorothea Fiedler

Point-by-point response to reviewers and Editor's Comments

Reviewer 1:
Recommendation: Reconsider after major revisions noted.

Comments:

In this manuscript, Trung et al. describe a powerful 2D BIRD-{1H-13C}HMQC NMR method to detect and quantify metabolic labelling of 13C-tagged inositol species from cellular extracts. The authors demonstrate that by comparing to 13C-1H signals of inositol phosphate (InsP) standards, the same chemical species can be quantified following incorporation into multiple cell lines without need for chromatography- or electrophoresis-based separation. The authors apply this technique to address the InsP6-dephosphorylation pathway that produces lesser-phosphorylated metabolites following phosphorylation by a distinct synthesis pathway, a process that is as yet poorly understood. They identify from cellular extracts four major InsPs including InsP6 and Ins(1,3,4,5,6)P5, both of which were previously established to be abundant InsP metabolites, as well as distinguish between enantiomers of several such metabolites. The development and validation of this method is, from a technical point of view, outstanding. As a method, the isotopically labelled substrates and NMR analysis is an impressive achievement that will be useful for the field and a great illustration of how synthetic and analytical chemistry can be combined to inform on cellular metabolic pathways.

We thank the reviewer for appreciating the work that went into developing this method.

To demonstrate the utility of this method in elucidating metabolic pathways, the authors focus on the multiple inositol polyphosphate phosphatase 1 (MINPP1) as a target enzyme to further characterize its role in dephosphorylating both InsP6 and Ins(1,3,4,5,6)P5. Using kinetic assays of purified MINPP1 and 13C-labelled substrates, the authors characterize the activity of the enzyme to generate new chemical species that can be tracked via NMR. They establish kinetic parameters for the various dephosphorylation steps they observe, including the generation of two mostly overlooked members of the InsP family whose appearance in cells is eliminated in MINPP1-/- cells. However, the biological significance of these data remains unclear, including the physiological relevance of the kinetics data as it would apply in vivo, the relevance of the subsequent metabolic flux analysis in wild type and MINPP1-/- cells, and the physiological importance of the two InsP species that the authors focus on. Some or all of these issues merit substantial expansion. Additional specific comments follow:

Major comments:

1. A major concern with the results presented in figures 5, 6, 7, S7, S8, and S9 is that the kinetic parameters laid out in figure 6B would suggest that any catalytic activity of

MINPP1 beyond the initial reactions with InsP5[2OH], InsP6, and possibly Ins(1,4,5,6)P4 are unlikely to be of biological significance. The rate constants for subsequent steps beyond those already characterized in the literature for MINPP1 drop off by multiple log folds, resulting in the reaction rates measured in vitro over several days presented in these figures. Given the tremendous spread of rate constants, it is incumbent on the authors to provide substantial additional data to support the model that MINPP1 is the master regulator of all of these metabolic steps. Put another way, it is challenging to conceive that multiple reaction sequences involving more than 30 chemically distinct conversions would be catalyzed entirely by one enzyme in a cellular context, especially when it takes multiple days for these sequences to achieve more than 50% conversion in vitro.

The reviewer raises several valid points. We agree that a number of the observed *in vitro* reaction rates are very low and – if similar in cells – likely not of biological relevance. Nevertheless, the *in vitro* dephosphorylation rates of $InsP_5$[2OH] and $InsP_6$ amount to 330 nmol/(min mg enzyme) and 3 nmol/(min mg enzyme), respectively. These numbers are consistent with literature values for MINPP1 isolated from mammalian liver tissue (211 and 12 nmol/(min mg enzyme), respectively) and validate MINPP1's annotated function (Nogimori, K.; Hughes, P. J.; Glennon, M. C.; Hodgson, M. E.; Putney, J. W.; Shears, S. B. *J. Biol. Chem*. **1991**, *266* (25), 16499–16506).
The dephosphorylation of Ins(1,4,5)P_3 as observed in the *in vitro* setting is most likely irrelevant in a cellular setting, as there are $InsP_3$ phosphatases that are known to possess much higher activity. Using the rate constants from Fig 6b, we can estimate that MINPP1 has an activity of 0.3 nmol/(min mg enzyme) towards Ins(1,4,5)P_3 (for comparison: OCRL V_{max} = 8000 nmol/(min mg enzyme), INPP5A V_{max} = 5300 nmol/(min mg enzyme), INPP5B V_{max} = 25000 nmol/(min mg enzyme); Zhang, X.; Jefferson, A. B.; Auethavekiat, V.; Majerus, P. W. *Proc. Natl. Acad. Sci*. **1995**, *92* (11), 4853–4856). We have included a discussion to the manuscript, to acknowledge these issues.

Considering the reviewers comments, and given that we observed strong inhibition of MINPP1 activity by $InsP_6$, we felt it was necessary to re-run the *in vitro* dephosphorylation reactions with $InsP_6$ as a substrate, at a lower $InsP_6$ concentration. We have now further probed this inhibition (also upon request of reviewer 2) and could show that the inhibition is likely substrate competitive. This data is now included as Figure S15 and discussed in the main text.
We next repeated the $InsP_6$ dephosphorylation reaction at a concentration of 50 μM $InsP_6$ and monitored the intermediates using NMR (Figure S14). While the reaction rate of the dephosphorylation of $InsP_6$ remains very similar (~4 μM/h) for both 175 μM and 50 μM substrate concentration, the subsequent reaction rates of the intermediates are drastically accelerated, in agreement with decreased substrate inhibition by $InsP_6$. Consequently, Ins(1/3,2)P_2 is observed as the major species, > 50%, after 16 hours (compared to ~70 hours previously). We have included this data as Figure S14 and also discuss this observation in the main text.

Whether MINPP1 indeed is responsible for the dephosphorylation of $InsP_6$ all the way down to $Ins(2,3)P_2$ in cells is an intriguing question. To further substantiate, that $Ins(2,3)P_2$ is indeed the predominant cellular $InsP_2$ species, we now also prepared $3[^{13}C_1]$*myo*-inositol and repeated the metabolic labeling of HEK293 cells (WT and $MINPP1^{-/-}$). The obtained spectra further corroborate and complement the previous findings made with $1[^{13}C_1]$*myo*-inositol. The results have been included into Figure 3 and in Figure S5. All data pertaining to the synthesis of $3[^{13}C_1]$*myo*-inositol has been added to the SI.
Based on the NMR and CE/MS data, we can say with certainty, that the formation of $Ins(2,3)P_2$ in cells is dependent on the presence of MINPP1, because $Ins(2,3)P_2$ is completely absent in the $MINPP1^{-/-}$ cells (see also response to the minor comment 2 below). Because $InsP_5[3OH]$ accumulates in $MINPP1^{-/-}$ cells, we further posit that MINPP1 is required for further dephosphorylation, at least for the immediate next step. Nevertheless, we cannot rule out that the activity of additional phosphatases further downstream contributes to the formation of $Ins(2,3)P_2$ and we have adjusted Figure 7d accordingly.

Given that MINPP1 is a phytase-homolog and phytases are well known to carry out many consecutive dephosphorylation reactions, it does not seem far-fetched to attribute multiple dephosphorylation steps to MINPP1 (Greiner, R.; Larsson Alminger, M.; Carlsson, N. gunnar; Muzquiz, M.; Burbano, C.; Cuadrado, C.; Pedrosa, M. M.; Goyoaga, C. J. Agric. *Food Chem*. **2002**, *50* (23), 6865–6870). It has been shown in past examinations of MINPP1 that it is indeed promiscuous and uses multiple different InsPs as substrates, dephosphorylating them at multiple possible positions. For example, Craxton et al. stated the following already in 1997 (Craxton, A.; Caffrey, J. J.; Burkhart, W.; Safrany, T. S.; Shears, B. S. *Biochem. J*. **1997**, *328* (1), 75–81.): "[…] we have obtained the first definitive evidence that a single enzyme is responsible for all the inositol polyphosphate catabolic activities associated with native MIPP, which has not previously been purified to homogeneity. That is, the 3-phosphates are specifically hydrolyzed from Ins(1,3,4,5)P4 and Ins(1,3,4,5,6)P5 […], the 6-phosphate is specifically removed from Ins(1,4,5,6)P4 […]. Despite these precise positional specificities, every phosphate on InsP6 is susceptible to hydrolysis […]." While our data now clearly indicate that the 2-position of $InsP_6$ is resistant to MINPP1-mediated dephosphorylation, we do agree with the promiscuous activity of MINPP1 described above.

Considering also that lack of MINPP1 is not embryonically lethal but, much the opposite, its absence in MINPP1-deficient mice leads to minor or no phenotypic deficits (as reported in ref. 16), the role the authors suggest for MINPP1 in figure 7D and in the text as a key metabolic enzyme carrying out several steps of InsP6 dephosphorylation does not seem to be supported by the evidence presented. An equally compelling alternative hypothesis is that other enzymes, including the unidentified InsP6 phosphatase redundant with MINPP1's activity on this substrate (pg.12, line 8), might be involved in subsequent dephosphorylations to generate the lower phosphorylated inositol species the authors observed. Thus, as presented, the study does not convincingly show concordance between

the in vitro kinetics and cellular knockout data, which are the two main pieces of data put forward to support the key model in figure 7.

The reviewer makes an interesting point. It is indeed true, that MINPP1 deletion in mice models is not lethal (Chi, H.; Yang, X.; Kingsley, P. D.; O'Keefe, R. J.; Puzas, J. E.; Rosier, R. N.; Shears, S. B.; Reynolds, P. R. *Mol. Cell. Biol*. **2000**, *20* (17), 6496–6507). Nevertheless, more and more phenotypes are now associated with loss of function of MINPP1 in mammals (apoptosis related to ER stress, and recently pontocerebellar hypoplasia in human patients with MINPP1-loss-of-function-mutations), some of them severe. Phenotypes can often also be conditional. That has not been explored systematically to date for MINPP1. We agree that the unidentified 3-phosphatase could potentially compensate for loss of MINPP1 (at least in part) in the mouse models displaying no or only subtle phenotypes, which was already suggested by Chi et al., who created these models. We have included a sentence in the manuscript to acknowledge this possibility. We are therefore heavily invested in determining the nature of this enzymatic activity in the future.

To accurately reflect these thoughts, we have expanded the discussion as follows:
"As MINPP1 is a homolog of phytases, which take part in inositol recycling/ scavenging, this possibility does not seem far-fetched. We cannot exclude the existence of other unknown phosphatases that contribute to this dephosphorylation pathway, however the accumulation of $InsP_5$[3OH] in MINPP1$^{-/-}$ cells suggests that MINPP1 is obligatory for the dephosphorylation of $InsP_5$[3OH]. Furthermore, the complete absence of Ins(2,3)P_2 in MINPP1$^{-/-}$ cells indicates that MINPP1 must carry out the key dephosphorylation of $InsP_6$ on the path towards Ins(2,3)P_2."

We would also like to point out that the goal of our manuscript was to report a powerful method for the analysis of complex InsP mixtures, for both *in vitro* and *in cellula* settings. The characterization on MINPP1 activity in this context emerged as an interesting application that uncovered several underappreciated features of this enzyme. It was not our intent to provide a full phenotypic characterization on MINPP1 within the scope of this manuscript.

"Thus, as presented, the study does not convincingly show concordance between the in vitro kinetics and cellular knockout data, which are the two main pieces of data put forward to support the key model in figure 7."

We respectfully disagree with this statement from the reviewer. Taking into consideration the data from the original submission, with the added experiments conducted for this re-submission, we feel that the concordance between *in vitro* dephosphorylation reactions, and the cellular knock out data is valid. We have unequivocally proven that the major $InsP_2$-species in mammalian cells is Ins(2,3)P_2, using asymmetrically labeled *myo*-inositol. While

the initial *in vitro* experiments with regards to $InsP_6$ dephosphorylation indeed seemed to indicate slow conversion to $Ins(2,3)P_2$, this is likely due to substrate inhibition. We could demonstrate (see comments above) that the dephosphorylation of $InsP_6$-derived intermediates is accelerated with lower substrate concentration. Furthermore, the unambiguous depletion of $Ins(2,3)P_2$ to undetectable levels in MS-based measurements, and the accumulation of $InsP_5$[3OH] in MINPP1$^{-/-}$ cells clearly shows the necessity of MINPP1 for the dephosphorylation of $InsP_6$ and $InsP_5$[3OH].

2. Literature reports discussing MINPP1 (e.g. ref. 16) describe the role of the ER localization of the enzyme in its substrate specificity and role in inositol phosphate metabolism. The current manuscript does not directly address (except in the discussion) the compartmentalization of MINPP1 and the potential impact it would have on availability of the various InsP species the authors describe as being substrates in the dephosphorylation scheme. Questions of how the InsP substrates reach MINPP1 and in what proportions would merit much further discussion and representation in the schematics of Figure 1A and 7D.

We agree with the reviewer, that the supposed compartmentalization of MINPP1 still poses a conundrum. To make this point clear from the beginning, we have included some additional background to the introduction. While early studies suggest MINPP1 to be localized to the ER, others have also shown alternative localizations to the Golgi, in lysosomes, or even secreted in exosomes (see references 28-31). We have attempted to adjust Figure 1a accordingly, but did not find it helpful, given these multiple proposed localizations. The localization of MINPP1 may also depend on the cell line under investigation.

We have therefore performed subcellular fractionation of HEK293 cells and checked for the localization of MINPP1 with organelle markers. The results suggest that MINPP1 indeed localizes to the ER. However, in addition, we also observe MINPP1 in mitochondria, but not in the nucleus. Furthermore, our data indicate that MINPP1 is predominantly soluble, and to a lower extent membrane-bound. We have included this data as Figure S7.

Given these results it is interesting to think about how substrate access to MINPP1 is regulated, in particular considering its proposed localization. Taking into account the strong inhibition of MINPP1 by $InsP_6$, MINPP1 would need to access localized pools of InsP substrates that are tightly regulated to either avoid or make use of the inhibitory effect. An intriguing avenue for regulation could be that MINPP1 remains localized to intracellular organelles (ER or lysosomes) into which InsPs are controllably translocated and then dephosphorylated. This dephosphorylation could potentially proceed all the way down to *myo*-inositol – with the aid of additional phosphatases - which could then be released through inositol transporters like SLC2A13 (HMIT). HMIT is known to be localized in intracellular membranes due to its ER-retention sequence and internalization sequence.

We feel that future experiments, in which the InsP composition of different organelles will be analyzed, will be of great use. Unfortunately, the current tools for such experiments are still the limiting factor.

We have included this discussion in the manuscript.

Minor comments:

1. Figure 3C only includes one replicate for HT29 cells. Three replicates should be included to be able to compare these results to those from the other cell lines.

We thank the reviewer for pointing this out. We have performed two additional biological replicates for the $[^{13}C_6]$*myo*-inositol-labeling of HT29 cells and included them in Figure 3C.

2. Figure 7B and 7C don't show all four metabolites in either model. The Ins(2,3)P2 in the MINPP1-/- cells would be especially useful for validating the data shown in figure 4B.

We thank the reviewer for raising this point. The amounts of Ins(2,3)P_2 in the MINPP1$^{-/-}$ cells, and the levels of InsP_5[3OH] in the WT cells, were below the limit of detection, despite the higher sensitivity of the mass spectrometry-based measurements. We therefore could not calculate percentage values and could not include these data in Figures 7B and C. We have now highlighted this observation in the caption of Figure 7. The fact that also CE-MS measurements could not detect the respective InsPs validates our conclusion from the reported NMR data. To make this point more clear, we modified the main text as follows: "Interestingly, in HEK293 MINPP1$^{-/-}$ cells, again, no Ins(2,3)P_2 was observed above the limit of detection, although the sensitivity of CE-MS is superior to NMR. Thus, CE-MS analysis confirms that generation of Ins(2,3)P_2 is dependent on MINPP1. Similarly, in the biosynthetic sequence InsP_5[3OH] is generated after InsP_6 (Figures 7c,d), hinting at an unidentified 3-phosphatase activity acting on InsP_6, which has been suggested in the past. Nevertheless, InsP_5[3OH] was not detectable in HEK293 WT cells."

Comments by Line:

1. Pg. 4 line 54, "labelled" misspelled
2. Pg. 6 line 44, "extracts" misspelled
3. Pg. 9 line 32, extra space between 3 and %

We have corrected these typing errors and thank the reviewer for the careful reading of the manuscript.

Reviewer 2:

Recommendation: Publish in ACS Central Science after minor revisions noted.

Comments:
The manuscript by Trung, et al. applies 13C-NMR probes to quantify the abundance of various inositol phosphates in multiple human cells lines. Inositol phosphates are ancient and extremely important signaling molecules present in all forms of life, from viruses to bacteria to plants and humans, and thus discoveries about such broadly important small biomolecules are of high impact and very broad interest. The authors here have made the unexpected discovery that several understudied forms of inositol phosphates are highly abundant in human cells, applying relatively new 13C-NMR metabolic labels to this long-standing problem. They go on to identify the enzyme responsible for the presence of these inositol phosphate species as MINPP1, using mammalian genetics, and elucidate the entire pathway for their synthesis by effectively applying computational metabolic flux analyses. The data strongly support all conclusions in the paper, and the conclusions do not overstate the data.

We thank the reviewer for the positive assessment.

Thus, this manuscript deserves publication after a few relatively minor problems have been addressed:

Major:

1) Kinetic analyses of MINPP1 use re-folded enzyme, given the unexpected nature of the IP6 curves some orthogonal confirmation of only the key aspects of these experiments using MINPP1 that has not been re-folded should be done. PTMs, etc, of eukaryotic sourced protein (if required) has its own caveats, but can also be denatured and refolded as a control.

We appreciate the reviewer's comment and certainly share these concerns.
As pointed out in the SI, we initially isolated lower amounts of recombinantly expressed MINPP1 from the soluble fraction of *E. coli* lysates, which was not subjected to refolding. Using this MINPP1 preparation (Sol.) as the non-refolded control, we have reassessed key aspects of the biochemical properties of MINPP1.

We found that both MINPP1 preparations [Sol. and from inclusion bodies (IB)] dephosphorylated $InsP_6$ *via* the same intermediates, in comparable amounts of time. This piece of information has been included as Figures S9a, b.

We have also compared reaction rates of Sol. and IB MINPP1 with three different substrates

($2,3$-bisphosphoglycerate, $InsP_5$[2OH] and $InsP_6$) using a Malachite-green assay kit (Sigma). Again, we observed similar reaction rates, with a maximal two-fold variation between Sol. and IB. We have included these experiments in the manuscript in Figure S9c. Also included is the literature value for the dephosphorylation of 2,3-bisphosphoglycerate of soluble recombinant MINPP1, as determined by Cho *et al*. (Cho, J.; King, J. S.; Qian, X.; Harwood, A. J.; Shears, S. B. *Proc. Natl. Acad. Sci. U. S. A.* **2008**, *105* (16), 5998–6003), which is very similar to our preparations. We therefore conclude, that the mild isolation from inclusion bodies does not significantly alter the activity of recombinant MINPP1, at least not with respect to the substrates under investigation.

With regards to post-translational modifications, we hypothesize that they only have a limited influence on the *in vitro* activity of MINPP1. Cho *et al*. (Cho, J.; Choi, K.; Darden, T.; Reynolds, P. R.; Petitte, J. N.; Shears, S. B. *J. Biotechnol*. **2006**, *126* (2), 248–259) have overexpressed the avian homolog of MINPP1 in yeast (*P. pastoris*) and it was subsequently treated with the commercial endoglycosidase Hf. Deglycosylation lowered the activity of MINPP1 by approximately 10%.

2) Fig 6 shows IP6 inhibits MINPP1, and the mechanism is quite rightly suggested to be substrate competitive, but this is not demonstrated. IP6 should be more thoroughly characterized as a substrate competitive, non-competitive or uncompetitive inhibitor of MINPP1 activity on InsP5[2OH] dephosphorylation. If IP6 acts as a non-competitive or uncompetitive inhibitor, the impact of the work gets much higher.

We thank the reviewer for sharing this interesting thought and we proceeded to investigate the mode of inhibition of MINPP1 by $InsP_6$. Due to the low K_M value of $InsP_5$[2OH] for MINPP1 (40 nM: Nogimori, K.; Hughes, P. J.; Glennon, M. C.; Hodgson, M. E.; Putney, J. W.; Shears, S. B. *J. Biol. Chem*. **1991**, *266* (25), 16499–16506), it was not possible to conduct Lineweaver-Burk-type experiments using NMR measurements, due to the relatively low sensitivity. We therefore explored alternative assays based on the detection of released inorganic phosphate (Pi). However, neither a commercial Malachite green assay kit (Sigma) nor a protein-based phosphate sensor (Invitrogen, PV4406) were sensitive enough to reliably detect < 20 nM Pi (the latter assay also showed cross-reactivity with buffer components). Without radiolabeled $InsP_5$[2OH], it was therefore not possible to conduct proper Lineweaver-Burk analysis.

Instead, we opted for a more qualitative assessment, and asked whether the Cheng-Prusoff equation applies to the reaction, which would confirm the competitive nature of the inhibition. If the substrate concentration is far greater than the K_M (e. g. 175, 70, and 20 μM $InsP_5$[2OH] *vs*. K_M = 40 nM) the IC_{50}-value for un- and non-competitive inhibition becomes mostly independent from the substrate concentration (Cheng, Y.-C.; Prusoff, W. H. *Biochem. Pharmacol.* **1973**, *22* (23), 3099–3108). We therefore measured the dephosphorylation of $InsP_5$[2OH] by MINPP1 at different substrate concentrations in the presence of $InsP_6$,

determined the corresponding IC_{50}-values and included the results as an additional SI Figure (Figure S15). The determined IC_{50}-values matched the predictions made by the Cheng-Prusoff equation well, and we thus conclude that the inhibition of $InsP_6$ against MINPP1 acting on $InsP_5$[2OH] is predominantly of competitive nature.

We have made the following adjustments to the main text and Figure 6C to accommodate these additional findings and to more correctly represent the reported IC_{50}-values:
"With changing substrate concentrations, the IC_{50}-value also changed as predicted by the Cheng-Prusoff equation, indicating that this inhibition is likely competitive or has a strong competitive component (Figure S15)." was added to the main text.
The reported IC_{50}-value in Fig 6C now reads "1.97 (±0.02)" and we expanded the caption to read "The IC_{50} value is reported with standard error of $log_{10}IC_{50}$ in brackets."

3) Strong, orthogonal data support that Ins(2,3)P2 is high abundance in the two cell lines examined, impact would be enhanced if other cells were tested (e.g. yeast, bacteria, sf9 lepidopteran or even primary human hepatocytes) to broaden this conclusion. This would also better demonstrate flexibility of the 13C-NMR tools.

We agree with the reviewer that the method should readily be applicable to other cell lines and other eukaryotic systems. In addition to HEK293 and HCT116 cells, our manuscript also reports the detection and quantification of different inositol phosphate species from HT29 (human colorectal adenocarcinoma), H1HeLa (cervical adenocarcinoma), and H1975 (lung adenocarcinoma) cells (Figure 3c and Figure S3), following metabolic labeling with [$^{13}C_6$]*myo*-inositol. Ins(2,3)P_2 was detected in all of them.
Following the reviewer's suggestion, we also submitted the yeast Schizosaccharomyces pombe (*S. pombe*) to metabolic labeling with [$^{13}C_6$]*myo*-inositol and have included the annotated spectrum as Fig S6. Interestingly, while $InsP_6$, Ins(1)P, Ins(2)P, and GroPI are readily detectable, Ins(2,3)P_2 does not appear to be present at sufficient concentrations. Instead, additional triplet signals outside of the clusters described in Fig. 2 are observed in the *S. pombe* extract, which indicates that *S. pombe* contains additional – so far not annotated - metabolites derived from *myo*-inositol. Annotating and probing the metabolism of these novel intermediates is an intriguing perspective for future work. We briefly refer to Figure S6 in the main text, but decided not to go into detail for the sake of clarity.

Minor:
4) In addition to reporting inositol phosphate concentrations using estimations of packed cell volumes, the authors should also report absolute quantifications to the internal standards somewhere in the paper.

We thank the reviewer for this suggestion. We have added an additional Supplementary file (SI_Cellular_quantification.xlsx) containing all raw data and calculations needed for determining the cellular InsP concentrations. The values in columns named "Concentration

in NMR sample" are absolute concentrations of InsPs measured in the NMR sample against the internal standard (TMPBr) prior to calculating back to packed cell volumes.

5) Figure 3 concentration values also must estimate number of cells/genomes in the sample in addition to cell volumes to estimate the cellular concentration. These numbers should be included in main text of Fig 3 and in the legend.

Given the inclusion of the supplementary file mentioned in the previous point (SI_Cellular_quantification.xlsx) we have added cell counts for the cell samples (note that HCT116 cells were not counted) in the supplementary file. However, the cell counts were not used for any calculations as we found over time that PCV provided the most reproducible results.

6) No details on the MINPP1-KO cell line could be found in the supplemental. The genetics here are important because HEK293 cells often have more than two copies of entire chromosomes. How was MINPP1-KO established (western, pcr, etc)? How many copies of MINPP1 are present in WT HEK293 cells? Did the CRISPR hit all the alleles? Importantly, this is only a minor concern because regardless of the ploidy the conclusions still hold - even if the cells are MINPP1 -/+/+, the phenotype suggests even a small reduction in MINPP1 alters the relevant InsPs. This just needs clarification.

We thank the reviewer for pointing this out. In our study we used the MINPP1$^{-/-}$ HEK293 cells that were described by Ucuncu *et al*. (Ucuncu, E.; Rajamani, K.; Wilson, M. S. C.; Medina-Cano, D.; Altin, N.; David, P.; Barcia, G.; Lefort, N.; Banal, C.; Vasilache-Dangles, M. T.; Pitelet, G.; Lorino, E.; Rabasse, N.; Bieth, E.; Zaki, M. S.; Topcu, M.; Sonmez, F. M.; Musaev, D.; Stanley, V.; Bole-Feysot, C.; Nitschké, P.; Munnich, A.; Bahi-Buisson, N.; Fossoud, C.; Giuliano, F.; Colleaux, L.; Burglen, L.; Gleeson, J. G.; Boddaert, N.; Saiardi, A.; Cantagrel, V. *Nat. Commun*. **2020**, *11* (1)). We have included the reference in the SI. Briefly, HEK293 cell lines were generated via a CRISPR-Cas9 genome editing strategy. SgRNAs targeting the first exon of MINPP1 transcript variant 1 (NM_004897.5) were designed on CRISPOR website (http://crispor.tefor.net/) and further cloned into the pSpCas9(BB)-2A-GFP plasmid (PX458, 48138, Addgene). For the generation of MINPP1$^{-/-}$ HEK293 clones, transfection of pSpCas9(sgRNA)-2A-GFP into HEK293 cells was performed with Lipofectamine 2000. Two days post transfection, single GFP+ HEK293 cells were sorted into 96-well plates by Fluorescence-activated cell sorting (FACS), Indel mutations of clones were detected by Sanger sequencing, and target editing efficiency was assessed by TIDE analysis.
In addition, we have also independently verified the absence of MINPP1 in MINPP1$^{-/-}$ HEK293 cells *via* Western Blot, which is included in Figure S7.

Editor Comments/Formatting Needs:

SI PG#S: The supporting information pages must be numbered consecutively, starting with page S1.

We have included page numbers as requested.

EMAIL: Please label as "email."

We have checked the email label.

GENERAL REF FORMATTING: Periodical references should contain authors' surnames followed by initials, article title, journal abbreviation, year, volume number, and page range. Refs with more than 10 authors should list the first 10 and then be followed by “et al.”
Web sources must include access date.

We have adopted the reference style as requested.

SYNOPSIS: Please label

The synopsis has been labeled.
